# Precision magnetic field modelling and control for wearable magnetoencephalography

**DOI:** 10.1016/j.neuroimage.2021.118401

**Published:** 2021-07-15

**Authors:** Molly Rea, Niall Holmes, Ryan M. Hill, Elena Boto, James Leggett, Lucy J. Edwards, David Woolger, Eliot Dawson, Vishal Shah, James Osborne, Richard Bowtell, Matthew J. Brookes

**Affiliations:** a Sir Peter Mansfield Imaging Centre, School of Physics and Astronomy, University of Nottingham, University Park, Nottingham, NG7 2RD, UK; b Magnetic Shields Limited, Headcorn Road, Staplehurst, Tonbridge, Kent, TN12 0DS, UK; c QuSpin Inc., 331 South 104th Street, Suite 130, Louisville, 80027, Colorado, USA

**Keywords:** Optically-pumped magnetometer, OPM, Magnetoencephalography, MEG, Magnetic field, Nulling

## Abstract

Optically-pumped magnetometers (OPMs) are highly sensitive, compact magnetic field sensors, which offer a viable alternative to cryogenic sensors (superconducting quantum interference devices – SQUIDs) for magnetoencephalography (MEG). With the promise of a wearable system that offers lifespan compliance, enables movement during scanning, and provides higher quality data, OPMs could drive a step change in MEG instrumentation. However, this potential can only be realised if background magnetic fields are appropriately controlled, via a combination of optimised passive magnetic screening (i.e. enclosing the system in layers of high-permeability materials), and electromagnetic coils to further null the remnant magnetic field. In this work, we show that even in an OPM-optimised passive shield with extremely low (<2 nT) remnant magnetic field, head movement generates significant artefacts in MEG data that manifest as low-frequency interference. To counter this effect we introduce a magnetic field mapping technique, in which the participant moves their head to sample the background magnetic field using a wearable sensor array; resulting data are compared to a model to derive coefficients representing three uniform magnetic field components and five magnetic field gradient components inside the passive shield. We show that this technique accurately reconstructs the magnitude of known magnetic fields. Moreover, by feeding the obtained coefficients into a bi-planar electromagnetic coil system, we were able to reduce the uniform magnetic field experienced by the array from a magnitude of 1.3 ± 0.3 nT to 0.29 ± 0.07 nT. Most importantly, we show that this field compensation generates a five-fold reduction in motion artefact at 0–2 Hz, in a visual steady-state evoked response experiment using 6 Hz stimulation. We suggest that this technique could be used in future OPM-MEG experiments to improve the quality of data, especially in paradigms seeking to measure low-frequency oscillations, or in experiments where head movement is encouraged.

## 1. Introduction

Magnetoencephalography (MEG) (Cohen, 1968) measures magnetic fields generated above the scalp by neuronal current flow in the brain. Mathematical modelling of these fields (or source reconstruction) forms 3D images showing moment-to-moment changes in brain electrophysiology. MEG offers a high spatial and temporal resolution assessment of neural activity (Baillet, 2017; Hämäläinen et al., 1993) in which the formation and dissolution of networks can be tracked in real time (Baker et al., 2014). Human brain dysfunction in neurological and psychiatric disorders can also be assessed, potentially offering powerful biomarkers of disease.

Despite its utility, the current generation of MEG systems is limited due to the use of superconducting quantum interference devices (SQUIDs) to detect the neuromagnetic field (Cohen, 1972). Whilst extremely sensitive, these cryogenic sensors must be fixed rigidly in position inside a liquid helium dewar, and the requisite thermally insulating vacuum space makes it difficult to position sensors closer than ~2 cm to the scalp. This reduces the strength of the MEG signal at the detectors (in accordance with an inverse square law), an effect which becomes further pronounced when scanning people with small heads (e.g. infants). It also limits our ability to sample the highest spatial frequencies of the magnetic field pattern (since field patterns become more spatially diffuse with distance). This, in turn, limits spatial resolution (Boto et al., 2016; Iivanainen et al., 2017). The fixed nature of the array also means that motion of a participant during a scan is restricted to less than 5 mm (Gross et al., 2013). Although small head motion (up to a few centimetres) inside the helmet can be algorithmically corrected (Nenonen et al., 2012; Taulu et al., 2005), large movements cannot be made, and no algorithms can correct for changing signal-to-noise ratio when a participant gets closer to or further from the sensors. This requirement to keep still over lengthy scans makes MEG inaccessible to many interesting study groups, and these confounds mean MEG has remained primarily a research tool, despite its clinical advantages over techniques such as electroencephalography (EEG) (Baillet, 2017; Boto et al., 2019).

Recent advances in quantum sensing have allowed the construction of ‘wearable’ or ‘on-scalp’ MEG systems where magnetic field sensors are placed either directly onto, or held near to the scalp. Optically-pumped magnetometers (OPMs) have emerged as the stand-out sensor technology in this area (Borna et al., 2020; Boto et al., 2021; Hill et al., 2020; Iivanainen et al., 2019), although arrays of High-Tc SQUIDs, which operate at liquid nitrogen temperatures, also offer great promise (Pfeiffer et al., 2019). Briefly (see Tierney et al., 2019 for a review), OPMs comprise a small, heated cell containing a vapour of alkali metal (e.g. rubidium) that is optically-pumped into a magnetically-sensitive state using laser light (Happer, 1972). Once prepared, the optical properties of the system (e.g. the transparency of the cell to the pumping laser) can be used to infer the magnetic field experienced by the atoms in the cell (Bell and Bloom, 1961, 1957; Dupont-Roc et al., 1969). For OPMs to attain sufficient sensitivity to detect changes in the neuromagnetic field, they are operated around a zero-field resonance in the spin-exchange relaxation free (SERF) regime (Allred et al., 2002). The ability of OPMs to measure neuromagnetic fields – first shown by Xia et al. (2006) – is now well established (e.g. (Boto et al., 2017; Johnson et al., 2010; Kamada et al., 2015; Sander et al., 2012)) and sensor miniaturisation and commercialisation (Allred et al., 2002; Johnson et al., 2013; Kamada et al., 2015; Kominis et al., 2003; Mhaskar et al., 2012; Sander et al., 2012; Schwindt et al., 2007; Shah and Wakai, 2013; Sheng et al., 2017) means that robust, small, and lightweight OPMs can now be mounted on the scalp surface. This allows for the introduction of wearable systems where the lightweight sensor array, mounted in a helmet, can be adapted to different head shapes/sizes and moves with the head during a scan (Boto et al., 2018; Hill et al., 2020). Given this, alongside lower overall cost, it is likely that OPMs will lift the significant limitations associated with cryogenic MEG systems. Challenges remain, however, before OPMs can replace SQUIDs as the fundamental building block of MEG instrumentation.

One of the biggest challenges in operationalising OPM-MEG is generating a well-controlled magnetic field environment. MEG systems (based on SQUIDs and OPMs) are housed inside magnetically-shielded rooms (MSRs). These are typically constructed from multiple layers of high magnetic permeability metal (e.g. mu-metal) to exclude low-frequency (DC to ~10 Hz) magnetic fields, and a layer of a metal with a high electrical conductivity (such as copper or aluminium) to attenuate higher-frequency (>10 Hz) magnetic fields (Hoburg, 1995). These ‘passively’ shielded enclosures provide screening of external sources of interference, which improve the signal-to-noise ratio of MEG data. However, the ferromagnetic properties of the high-permeability materials used in MSR construction often result in a ‘remnant’ DC (i.e. temporally static) magnetic field. SQUIDs are insensitive to such DC fields, but OPMs (being *zero-field* magnetometers) are very sensitive to background magnetic fields and fail to operate in fields larger than a few nT. For this reason, OPMs typically come equipped with on-board electromagnetic coils that null the local magnetic field vector experienced by the cell. In open-loop mode, currents in these coils are set at the beginning of an experiment, meaning that sensors can be rendered inoperable by field changes produced when the OPMs move during acquisition (i.e. if head movement occurs when using a wearable system). For example, in a 30 nT background magnetic field, a rotation of ~4° would generate sufficient change in field to take an OPM outside its ±1.5 nT dynamic range (defined here as a 5% gain error) (Boto et al., 2018; Iivanainen et al., 2019). Even if the sensor were to remain working, the resulting magnetic artefact would be much larger in amplitude than the neuromagnetic field. For these reasons, suppressing background static magnetic field – even beyond what is achieved with passive shielding – is critical for OPM-MEG.

In previous work (Holmes et al., 2019, 2018), we designed and constructed a bi-planar electromagnetic coil system that contains multiple coil elements, each designed to produce a distinct, homogeneous, component of either magnetic field or magnetic field gradient over a 40 × 40 × 40 cm^3^ volume at the centre of the two planes. The remnant magnetic field inside a MSR was compensated by computing estimates of the magnetic field (and its gradients) using a fixed OPM reference array. These estimates were fed into independent feedback controllers to automatically drive field estimates to zero by varying the currents in the bi-planar coil elements. Field mapping before and after the cancellation revealed a reduction in the magnitude of the magnetic field from 28 nT to 0.74 nT. This, in turn, enabled participant movements (Holmes et al., 2018). Dynamic compensation of large sources of interference below 1 Hz has also been demonstrated using a high-speed feedback controller to modulate the coil currents (Holmes et al., 2019). These bi-planar coil systems have proved effective, allowing OPM-MEG studies to be carried out in freely moving participants (e.g. (Hill et al., 2019; Roberts et al., 2019)). However, accurate assessment of the background magnetic fields is limited by the geometry of the reference array, and significant improvements could be made if we were able to more accurately model the remnant magnetic field that informs the optimal coil currents.

Recently, a new MSR dedicated to OPM measurements has been installed at the Sir Peter Mansfield Imaging Centre, University of Nottingham. This room comprises four layers of mu-metal and one layer of copper, and features demagnetisation (or ‘degaussing’) coils wound around the mu-metal panels (MuRoom, Magnetic Shields Ltd., Kent, U.K.). When driven with a linearly decaying sinusoidal current, these coils force the mu-metal around its hysteresis curve towards a point of zero magnetisation (Altarev et al., 2015, 2014; Voigt et al., 2013). However, the effect of joints between mu-metal panels, the need for access apertures, and variations in the permeability of the mu-metal mean that a remnant magnetic field of around 2 nT still remains. Nevertheless, this is a significant improvement over the ~30 nT remnant magnetic field present within another MSR at our institution that houses a cryogenic MEG system (for comparison, this room consists of two layers of mumetal, one layer of aluminium and does not feature degaussing coils (Vacuumschmelze, Hanau, Germany)). A remnant magnetic field of 2 nT allows OPMs to operate with minimal compensation currents applied to their on-sensor coils. Despite this improvement, full rotation of the head (e.g. during ambulatory motions) would produce a field shift that would render an OPM inoperable. Smaller movements that do not saturate OPM outputs will still generate magnetic field artefacts in MEG data that would mask brain activity (particularly at low frequency). For these reasons, integrating field compensation coils with the new generation of MSR remains of critical importance.

In this paper, we propose a new approach to using bi-planar coils in an OPM-optimised MSR. Starting with a magnetic field of <2 nT, we combine optical tracking of the movements of an OPM-array worn by a participant with synchronised recording of magnetometer data to generate a model of the remnant magnetic field inside the MSR. Compared to our previous approach using a reference array (Holmes et al., 2018), this model increases the number of components of magnetic field and field gradient that can be measured from six to eight, without surrounding the participant with additional reference sensors. A map of the magnetic field in the full volume of the head-mounted OPM array is produced, rather than at a limited number of sample points located a small distance from the participant. By combining the model with dynamic compensation of low-frequency interference and appropriate coil calibration, we were able to accurately null the background magnetic field inside the MSR. We begin by outlining the theory underlying the new field nulling method and describing our implementation. We then investigate the ability of this method to map known magnetic fields, and assess its performance when compensating the remnant magnetic field within our MSR. Finally, we present a MEG demonstration featuring continuous head-movements and compare sensor-level data analysis with and without magnetic field compensation.

## 2. Theory

We begin by assuming that the area inside the MSR is a current-free space and consequently we model the remnant magnetic field, ***B***(*x, y, z*) using a magnetic scalar potential Φ(*x, y, z*), thus

(1) B(x,y,z)=−∇Φ(x,y,z).

We set

(2) $$\nabla^{2}\Phi (x,y,z)=0,$$

in order to ensure that the magnetic field obeys Maxwell’s equations for magnetostatics in a current-free region; i.e.

(3) ∇⋅B(x,y,z)=0,

(4) ∇×B(x,y,z)=0.

As the scalar potential obeys Laplace’s equation, its solutions can be represented as the real spherical harmonics, where *n*^*th*^ order terms in the scalar potential generate *(n−1)*^*th*^ order terms in the magnetic field. We assume, for simplicity, that the local magnetic field at a position in or around the centre of the MSR, which is far from any sources of magnetic field, can be approximated as the sum of three spatially uniform components ***B***_***U***_, and five (linearly) spatially dependent magnetic field gradient components ***B***_***G***_, thus

(5) $$B_{local}(x,y,z)=B_{U}+B_{G}(x,y,z).$$

In the spherical harmonic field model, the 1st order terms in the scalar potential describe the three spatially uniform magnetic field components:

$$\text{uniform}\;\text{field}\;\text{components}\;\{\begin{array}{ll} B_{x}=\alpha_{1}\hat{x}, & \left\{B_{x}\right\}\\ B_{y}=\alpha_{2}\hat{y}, & \left\{B_{y}\right\}\\ B_{z}=\alpha_{3}\hat{z}, & \left\{B_{z}\right\}, \end{array}$$

and the 2nd order terms of the scalar potential describe the five magnetic field gradient components:

$$\{\begin{array}{l} G_{xy}=\alpha_{4}(y\hat{x}+x\hat{y}),\left\{\frac{dB_{x}}{dy}=\frac{dB_{y}}{dx}\right\}\\ G_{xz}=\alpha_{5}(z\hat{x}+x\hat{z}),\left\{\frac{dB_{x}}{dz}=\frac{dB_{z}}{dx}\right\}\\ G_{yz}=\alpha_{6}(z\hat{y}+y\hat{z}),\left\{\frac{dB_{y}}{dz}=\frac{dB_{z}}{dy}\right\},\\ G_{zz}=\alpha_{7}(-x\hat{x}-y\hat{y}+2z\hat{z}),\left\{2\frac{dB_{z}}{dz}=-\frac{dB_{x}}{dx}-\frac{dB_{y}}{dy}\right\}\\ G_{xx}=\alpha_{8}(x\hat{x}-y\hat{y}),\left\{\frac{dB_{x}}{dx}=-\frac{dB_{y}}{dy}\right\} \end{array}$$

where the coefficient, *α*_*n*_, describes the strength of the n^th^ component and $\hat{x}$, $\hat{y}$ and $\hat{z}$ are the Cartesian unit vectors. The equivalent magnetic field or magnetic field gradient for each term is shown in brackets. We then re-write Eq. (5) in terms of the *α*_*n*_ coefficients,

(6) $$B_{local}(x,y,z)=\alpha_{U}+G(x,y,z)\alpha_{G},$$

where ***α***_*U*_ contains the three uniform field coefficients, ***α***_*G*_ contains the five field gradient coefficients and ***G***(*x, y, z*) is a gradient characterisation matrix. Incorporating the expressions from the above model and using a column vector to represent the field, Eq. (6) becomes

(7) $$B_{local}(x,y,z)=\left(\begin{array}{l} B_{x}(x,y,z)\\ B_{y}(x,y,z)\\ B_{z}(x,y,z) \end{array}\right)=\left(\begin{array}{c} \alpha_{1}\\ \alpha_{2}\\ \alpha_{3} \end{array}\right)+\left(\begin{array}{ccccc} y & z & 0 & -x & x\\ x & 0 & z & -y & y\\ 0 & x & y & 2z & 0 \end{array}\right)\left(\begin{array}{c} \alpha_{4}\\ \alpha_{5}\\ \alpha_{6}\\ \alpha_{7}\\ \alpha_{8} \end{array}\right).$$

OPMs used in MEG are vector (as distinct from scalar, or total field) magnetometers that measure the magnetic field component along at least one sensitive axis. The component of magnetic field measured by an OPM at position (*x, y, z*) can be calculated as

(8) $$b_{e}(x,y,z)=B_{local}(x,y,z)\cdot e,$$

where ***e*** is a unit vector characterising the orientation of the sensitive axis of the magnetometer with respect to the MSR (i.e. $e=e_{x}\hat{x}+e_{y}\hat{y}+e_{z}\hat{z}$ and |***e***| = 1). From Eqs. (7) and 8 we note that rotation of a magnetometer about $\hat{x}$, $\hat{y}$ and $\hat{z}$ will generate a change in the measured field due to the uniform field components. Similarly, translations (and rotations) of the sensor will generate a change in the measured field due to the spatially dependent field gradient components.

By recording the initial positions and orientations of an array of OPMs, and then measuring changes in these positions and orientations as the array is rotated about all axes and translated in all directions, we can use Eqs. (7) and 8 to simulate the change in magnetic field that would be measured by the array, if the remnant magnetic field in the MSR through which the array was moved comprised a unit (e.g. 1 nT or 1 nTm^−1^) contribution of one of the eight coefficients. This can be repeated for all coefficients, and through comparing the simulated sensor time-courses with magnetic field data recorded simultaneously with the movement data, we can recover the coefficients that best approximate the remnant magnetic field. Mathematically, we generate a linear matrix equation:

(9) $$B_{\text{mat}}\alpha =b,$$

where ***α*** is a column vector that contains all eight coefficients, **B**_mat_ is a matrix characterising the fields that would be produced over the array of *N* OPMs by the eight unit-weighted field components, based on the sensor positions and orientations measured at each of *T* time-points (see Appendix A). **B**_mat_ has 8 columns (one for each field component) and *T* · *N* rows (one row for each sensor at each time-point at which the magnetic field and the sensor positions and orientations were measured). The target field column vector ***b*** contains the magnetic fields measured by each of the *N* OPMs at *T* time-points, at the same positions and orientations as in **B**_mat_. The coefficients ***α*** that produce the best fit to ***b*** can be obtained by identification of a pseudo-inverse matrix, or similar process.

In order to use the field model to then compensate the remnant magnetic field, currents need to be chosen in a series of electromagnetic coils, which produce an equal and opposite magnetic field to that found by the model. We assume, for now, that our coil system features eight distinct coils that each produce a single, spatially homogeneous component of the magnetic field or magnetic field gradient over the region in which the field was mapped. The magnetic or magnetic field gradient strength produced per unit of applied current can be matched to the negative of the model coefficients to generate appropriate currents. Note here that this argument necessarily assumes that the magnetic field inside the MSR is temporally stable. In practice, particularly for the low magnetic fields inside the OPM-optimised shield, this is unlikely to be the case due to external sources of interference, or slow temperature changes affecting the magnetisation of the mu-metal used to construct the MSR. For this reason, implementing high-speed dynamic stabilisation of the remnant field prior to the mapping procedure is crucial to enable accurate field mapping and compensation.

## 3. Experimental implementation

### 3.1. System overview

**Fig. 1.**
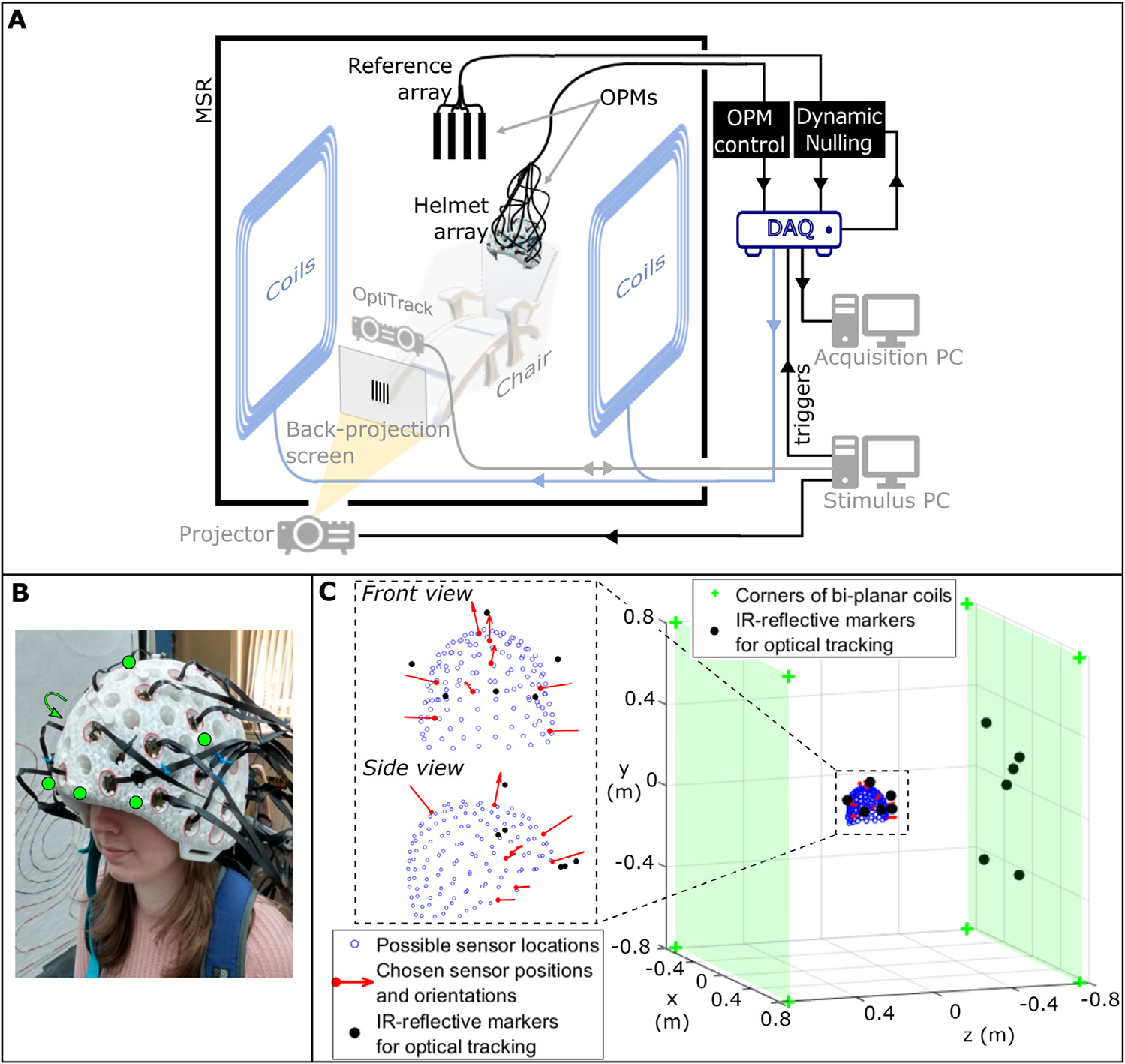
System overview. A) A schematic diagram of the OPM-MEG system at the University of Nottingham. B) The additively-manufactured rigid helmet used to mount the OPM sensors close to the scalp. Six IR-reflective markers are attached to the helmet at known locations to facilitate 6-DoF optical tracking (marked in the photograph with the green circles (an arrow indicates the sixth marker is positioned on the side of the helmet not visible here)). C) Location of the helmet between the bi-planar coils. The coils are shown by the green shading; the coil corners by the green crosses. The blue markers show OPM locations and the black dots show OptiTrack markers (on the helmet and coils). Inset, the helmet is shown with the location and sensitive orientation of the 8 OPMs used for collecting the data to fit to the field model. (For interpretation of the references to colour in this figure legend, the reader is referred to the web version of this article.)

To perform field mapping, we used an OPM-MEG device developed at the University of Nottingham and described in detail by Hill et al., 2020 (see Fig. 1A). In brief, the system comprises an array of up to fifty second-generation QuSpin Inc. (Louisville, Colorado, U.S.A.) zero-field magnetometers (Osborne et al., 2018) housed within a custom-built, additively manufactured helmet (Added Scientific Ltd., Nottingham, U.K.) (Fig. 1B). The additive manufacturing process means that the OPM locations and the orientations of the sensitive axes, relative to the helmet, are known accurately. Whilst in principle each OPM provides two sensitive axes, only fields orientated (approximately) radial to the head were measured. The magnetometer data were recorded using a National Instruments (NI, Austin, Texas, U.S.A.) digital acquisition system (DAQ), controlled using LabVIEW (NI, Austin, Texas, U.S.A.) at 16-bit resolution and a sampling rate of 1200 Hz. The system is housed within an OPM-optimised MSR (MuRoom, Magnetic Shields Ltd., Kent, U.K.), which provides an open scanning environment. The participant was positioned such that their head was within the central region of the room, where the remnant magnetic field and magnetic field gradient are <2 nT and <2 nTm^−1^ following degaussing (Altarev et al., 2015; Hill et al., 2020).

#### Bi-planar coils

The two planes of the bi-planar coil system (Holmes et al., 2018) were placed on either side of the participant (Fig. 1C). The system in use consists of seven distinct coil elements each designed to generate one of *α*_1_ to *α*_7_ in the magnetic field model (note that a coil corresponding to the eighth component in our model, i.e. *α*_8_, was unavailable at the time of these experiments). The coil planes are separated by 1.5 m, such that the head is located within the central volume. Coil currents are controlled using a voltage generated by a 16-bit digital-to-analogue converter interfaced with the DAQ via LabVIEW. These voltages are passed through a series of low-noise coil drivers provided by QuSpin *(*http://quspin.com/low-noise-coil-driver/) before being sent to the bi-planar coils. A resistor is added in series with the coils to ensure the magnetic field generated per unit voltage is within an appropriate range of approximately ±1 nTV^−1.^ This gives rise to ~0.25 fT magnetic field noise, to which the OPMs are insensitive.

#### Dynamic stabilisation

**Fig. B1.**
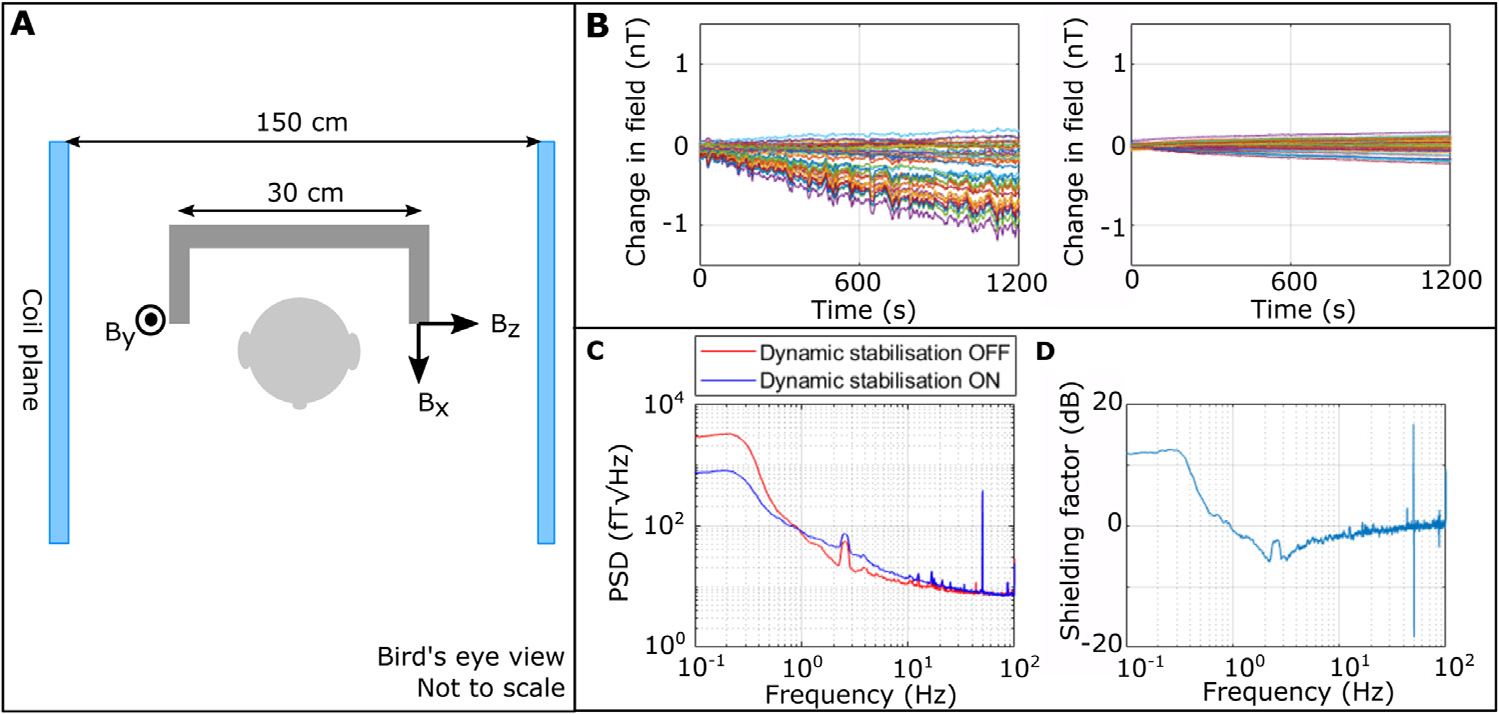
Demonstration of dynamic stabilisation. A) Experimental set up: The helmet was placed at the isocentre of the coils (and approximately at the centre of the MSR). The reference array is placed slightly off-centre, behind the helmet, to avoid collisions during mapping movements. Reference OPMs used for dynamic nulling are mounted either side of the head and separated by a distance of ~30 cm. B) Time-courses derived from 40 OPMs inside an empty MSR, without dynamic stabilisation (left) and with dynamic stabilisation active (right). C) Median (over sensors) power spectral density with (blue) and without (red) stabilisation. D) Estimated field attenuation of the system as a function of frequency (in decibels). (For interpretation of the references to colour in this figure legend, the reader is referred to the web version of this article.)

A reference array, comprising three, first-generation QuSpin OPMs, was placed in a fixed, orthogonal arrangement behind the participant’s head, more than 10 cm from the helmet to prevent any collisions during mapping movements (see Figure B1A). In previous work, this reference array was used to map the remnant magnetic field; however, this method was limited in terms of the number of spatial field components that could be calculated, and the accuracy of the resulting values. Here, instead, we used the reference array for dynamic stabilisation of changes in the uniform components of the remnant magnetic field. Outputs of the reference magnetometers were input to a high-speed (60 Hz), PI controller implemented in LabVIEW, which calculates compensation currents that are fed back to the bi-planar coils. This, in turn, generates temporally changing fields that dynamically compensate <3 Hz changes in the three uniform components of the magnetic field (Holmes et al., 2019). In practice, the user specifies a desired ‘set-point’ at which the OPM outputs are held. This is unlikely to be zero-field, but the process acts to stabilise the uniform background magnetic field to prevent low-frequency changes that would otherwise cause the field to ‘drift’ over time, and so reduce the accuracy of our mapping and degrade the quality of the nulling (see Appendix B).

#### Head tracking

Tracking of participants’ head motion was performed using an OptiTrack V120:Duo camera (NaturalPoint Inc., Corvallis, Oregon, U.S.A.), which facilitates six degrees of freedom (6-DoF) tracking of the centre of mass of a series of infra-red (IR) reflective markers, to sub-millimetre and sub-degree precision at a frame rate of 120 Hz. Motion tracking data were recorded using the NaturalPoint Motive software platform. Control of Motive from MATLAB (MathWorks Inc., Natick, Massachusetts, U.S.A.) was facilitated by the NaturalPoint NatNet SDK software. Six IR-reflective markers were attached to known locations on the OPM-MEG helmet to facilitate tracking of head movements and, by extension, sensor movements (since the locations of the sensors relative to the helmet are known from the additive manufacturing process). Six additional markers were added to one of the bi-planar coils to construct a stationary plane of reference (see Fig. 1C). The OptiTrack camera was positioned such that all twelve markers were visible when the participant was facing the camera. These two groups of six markers were then used to form rigid bodies, enabling tracking. During participant movement, the helmet rigid body could be accurately tracked by the Motive software provided at least three of the six markers were visible.

#### Data collection

Eight, head-mounted sensors, roughly positioned at the front, left, right, and top of the helmet (see inset diagram of Fig. 1C), were chosen to sample the background magnetic field. These OPMs were ‘field-zeroed’ (the process in which the on-sensor coils within each OPM are used to zero the field across its vapour cell) and calibrated after dynamic stabilisation of the background magnetic field had been initiated.

The participant was instructed to move their head for 60 s in order to rotate and translate the head-mounted sensors about and along three orthogonal axes, to ensure comprehensive sampling of each of the magnetic field components. The magnetometer and motion tracking data were simultaneously collected and synchronised using a DAQ trigger channel controlled by MATLAB, to indicate that Motive had started recording.

Two participants took part in this study. Both gave written informed consent, and the study was approved by the University of Nottingham Medical School Research Ethics Committee. Data are available upon reasonable request from the corresponding author.

### 3.2. Field characterisation using the model

The above process results in two separate (synchronised) datasets; magnetic field data recorded by the OPMs, and the movement data recorded by the OptiTrack camera. We used an affine transform to define the location and movement of the helmet in the coordinate frame of the bi-planar coils (which itself was made possible by the markers on the coil plane). The field model was also generated in this same frame of reference. In the motion data, there were a small number of frames in which the cameras failed to record the position of the helmet. Since a minimum of three markers must be visible to enable optical tracking, if the cameras lost sight of four of the six helmet markers due to the limited field of view of the OptiTrack Duo within the MSR, the rigid body position could not be tracked and an ‘empty frame’ was recorded in the motion data. In such empty frames, missing data were replaced by equivalent data from the previous frame.

Magnetometer field measurements were defined relative to the value measured at the start of the experiment (i.e. we subtracted the magnetic fields measured at the first time point from all subsequent time points). This was to ensure that the measured data represent the change in magnetic field due to the measured motion. Magnetometer data were down-sampled to 120 Hz to match the sampling frequency of the motion data. Both the magnetometer and motion datasets were then low-pass filtered using a finite impulse response filter of order 500, with a cut-off frequency of 10 Hz. (We assumed that motion would be in the 0–10 Hz range.)

Once processed, the magnetometer and motion data were used to construct the linear matrix equation characterised by Eq. (9). The motion data were used in Eqs. (7) and (8) to calculate **B**_mat_, which contains the simulated change in magnetic fields from unit-weighted field components over time caused by the changes in position and orientation. The processed magnetometer data were used to form the target field vector ***b***, and the coefficients of the magnetic field model, ***α***, that best map **B**_mat_ to ***b*** were obtained by calculation of the pseudo-inverse of **B**_mat_. All calculations were implemented using bespoke code in MATLAB. The resulting parameters completely describe the spatially uniform and linear gradient terms of the temporally static (i.e. DC) magnetic field. Recall that any drift (over time) is already accounted for by the dynamic stabilisation process, meaning that removal of this DC component should leave the system sitting in a zero magnetic field environment.

### 3.3. Field nulling

In order to null the remnant magnetic field, we need to feed the corresponding coefficients, calculated using the model, into the bi-planar coil system to generate a magnetic field equal and opposite to that measured. In theory, appropriate coil currents could simply be estimated from the known coil efficiencies. However, in practice, the presence of high-permeability materials used in the walls of the MSR distort the spatial variation of the fields produced by the coils and a failure to take this into account would lead to sub-optimal nulling. For this reason, we used a data-driven approach in which the strength and spatial profile of the (distorted) magnetic field patterns produced by the coils, over the array of OPMs, was measured. This was then used to inform our choice of coil currents.

We defined a coil calibration matrix ***C***, formed of 7 columns (one column per coil element) and 8 rows (one row per magnetic field component, *α*_1_ through *α*_8_). The elements of this matrix characterise the amount of each of the eight components of magnetic field that is generated by a unit current applied to each coil. The coil calibration matrix was defined *a priori*, using 47 OPMs to measure the change in magnetic field produced by a series of known currents applied to the seven coil elements. The OPMs were positioned within the rigid helmet to provide comprehensive coverage, and the helmet itself was placed within the central volume of the coils and MSR, where the head of the participant would be located. As before, the position of the helmet relative to the bi-planar coils was recorded using the OptiTrack camera. The measured magnetic field data at the known, fixed, sensor positions and orientations generated by coil currents were then fitted to our magnetic field model to calculate the elements of ***C***.

Following calculation of the coil calibration matrix and the identification of the remnant magnetic field coefficients via the field mapping process, a linear matrix equation

(10) $$Ci=-\alpha_{t},$$

is generated to find optimal coil currents for nulling. Here, ***i*** is a column vector containing the 7 coil currents and ***α***_***t***_ is a column vector containing the 8 coefficients found following field mapping. Coil currents that best produce the required magnetic field components are found by identifying the pseudo-inverse of the matrix ***C***. Note that we have an underdetermined system since our coil array is missing one element; in order to ensure optimal compensation of the 7 components for which we do have a distinct element, we artificially set the value of *α*_8_ in ***α***_***t***_ to zero (in order to avoid small contributions to *α*_8_ from each coil element impacting the calculation). To null the remnant magnetic field, dynamic stabilisation was briefly paused and coil currents held at the final value computed by the controller. The currents calculated via Eq. (10) were then added to the dynamic stabilisation currents to remove the DC component. Dynamic stabilisation was then reinitiated. This process takes <1 s and so field drift in this short time-window was minimal.

### 3.4. Overview

**Fig. 2.**
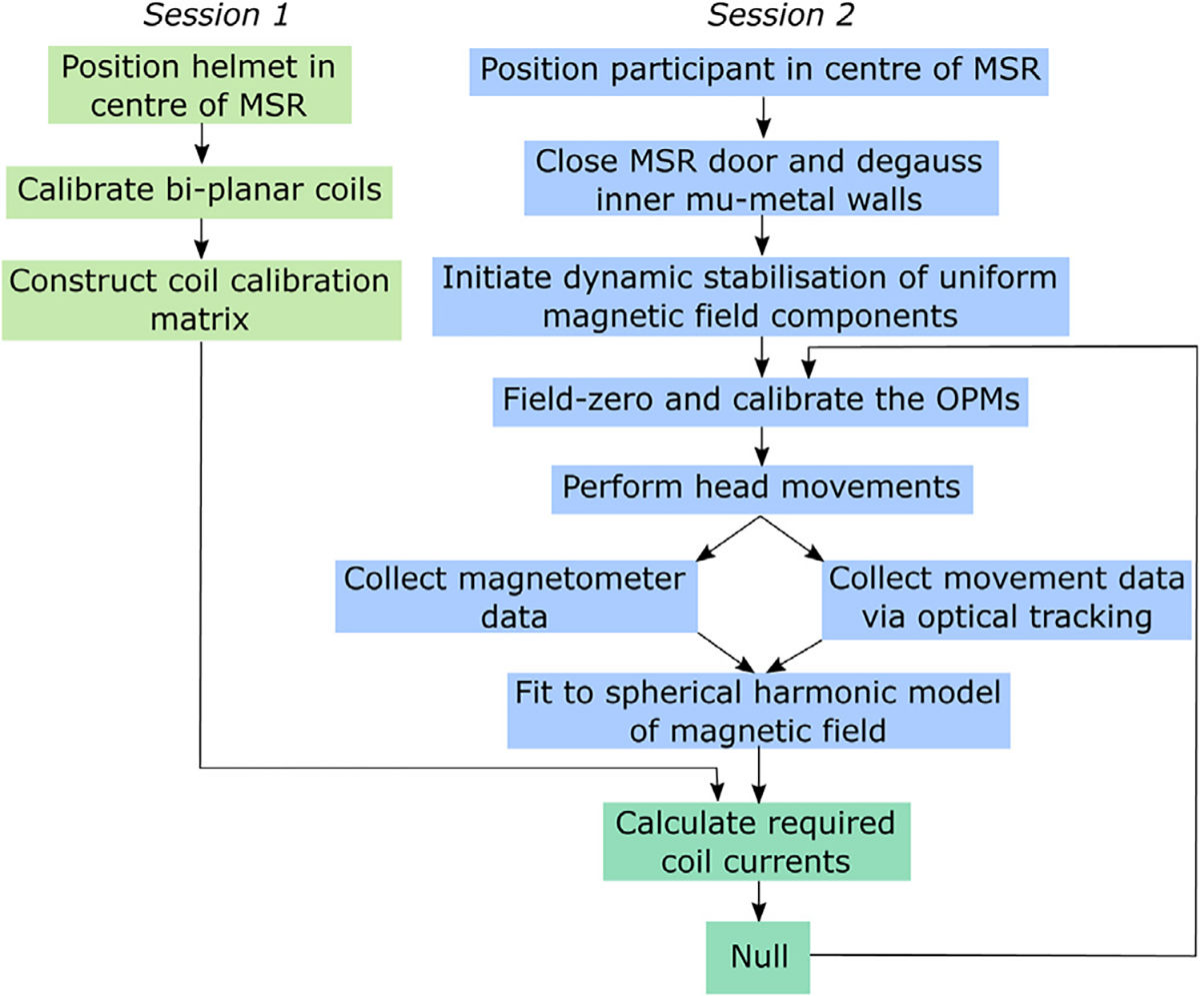
Schematic overview of the field nulling process.

An overview of the entire process is given in Fig. 2. The field nulling procedure itself takes approximately 4 min to carry out, including 1 min to record the data, and a further 3 min to complete the fit and feed the resulting data back to the bi-planar coils.

## 4. Mapping known fields

In our first experimental demonstration, we aimed to determine whether the model could accurately compute the known magnitude of a background field change that was deliberately applied inside the MSR.

### 4.1. Method

A single participant took part in this experiment. The additively manufactured helmet containing 40 OPMs was mounted on the head. All sensors were switched on and functioning, though only eight OPMs were involved in the field mapping process (as described in Section 3.1), as this was sufficient to map the remnant magnetic field comprehensively while minimising the time taken to compute model coefficients. The participant was seated with their head positioned approximately centrally in the MSR and at the isocentre of the bi-planar coils. The MSR door was closed and the internal mu-metal walls of the MSR degaussed. Dynamic stabilisation was applied and the background magnetic field was mapped via the procedure outlined in Section 3. To sample the background magnetic field, the participant moved their head such that they completed rotations about each Cartesian axis in turn, followed by translations along each axis. This series of movements was performed twice within the 60 s recording. Once the sequence of movements had been completed, the coefficients that described the remnant magnetic field and its linear gradients were calculated.

Following the initial field map, an offset magnetic field or magnetic field gradient was applied using a single element of the bi-planar coils: the coil current required to generate the desired magnetic field, or magnetic field gradient, was calculated using the appropriate diagonal element of the coil calibration matrix ***C***. Once the offset had been applied, the field mapping procedure was repeated. This process was repeated for each of the seven components in the field model for which we have dedicated coils, using offsets of 0.5 and 1 nT or nTm^−1^. For each component, and field strength, we repeated the experiment three times. Field offsets were determined by subtraction of the model coefficients calculated before and after the offset was applied. All values were averaged over the three repeat runs and the standard deviation computed.

### 4.2. Results

**Fig. 3.**
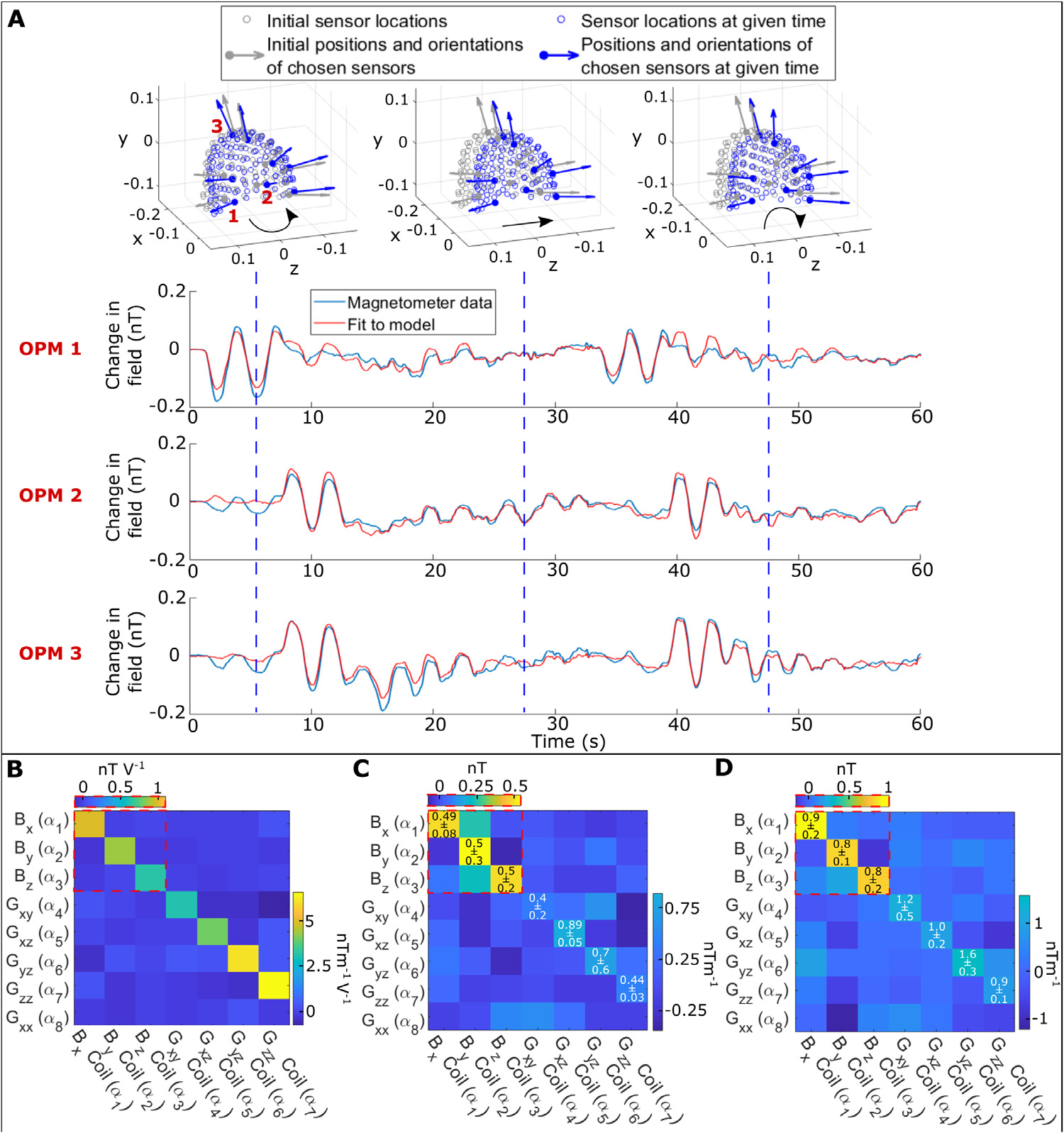
Mapping known fields. A) Representative data taken from three OPMs during a sequence of head movements. The blue line shows the magnetometer data. The red line shows the model fit. The three inset images show the position of the sensors (blue) relative to their initial position (grey) at three moments in time during the sequence of movements. B) Coil calibration matrix. Values represent the effect of pulsing each of the 7 available coils with a unit voltage. Notice that for visualisation, values for uniform magnetic field and field gradient are shown on different scales. As expected, each coil largely affects a single component of the model. C) The results of mapping known fields: a known magnetic field of 0.5 nT, or a field gradient of 0.5 nTm^−1^ was generated by each coil. The values in the matrix show the model fit to those fields. The numbers show how accurate the model fit was in terms of mean and standard deviation across runs (i.e. numbers along the diagonal should be close to 0.5). D) Equivalent to (C) but for known fields/gradients of 1 nT and 1 nTm^−1^ (numbers along the diagonal should be close to 1). See also Supplementary Material Figure S1. (For interpretation of the references to colour in this figure legend, the reader is referred to the web version of this article.)

Data for a single representative run are shown in Fig. 3A. Here, the line plots show magnetometer data (in blue) measured during the sequence of participant movements. The fit to these fields, based on the motion tracking data and field model, is shown in red. Notice that in this case the model fits well to the data, suggesting that the magnetometer data are dominated by field changes due to movement (as distinct from other low-frequency magnetic artefacts (see Discussion)). The three inset plots show the locations of the OPM array (in blue) relative to its location at the start of the experiment (in grey), at three different time-points during the sequence.

Fig. 3B shows the coil calibration matrix ***C***, in units of nTV^−1^ or nTm^−1^V^−1^ (note that in the matrix, for visualisation, different scales are used for components of the matrix that relate to uniform magnetic fields and magnetic field gradients). The matrix depicts the field generated at the helmet by pulsing a coil with a unit voltage generated by the DAQ. As expected, each coil maps almost exclusively to a different component in our field model, but with some small, off-diagonal elements that are likely caused by field interactions with the MSR (Holmes et al., 2019).

Fig. 3C and D show results of mapping known field offsets via our head movement-based mapping procedure. Fig. 3C shows results for a field of 0.5 nT or gradient of 0.5 nTm^−1^. Fig. 3D shows results for a field of 1 nT or gradient of 1 nTm^−1^. In both cases, the diagonal elements of the matrices are broadly consistent with the expected size of the applied magnetic field, showcasing the accuracy of the mapping procedure. In general, the accuracy of the uniform field components was higher than the accuracy of the gradients; this, we believe, is due to the scale of the applied fields. For example, a uniform magnetic field of 0.5 nT would generate a measured OPM signal change of order ~0.1 nT for the scale of motion carried out by the participant (e.g. a 10° head rotation). However, a magnetic field gradient of 0.5 nTm^−1^, would only produce a signal change of ~0.01 nT over the scale of motion carried out (e.g. a head translation of 2 cm). This means that field gradients are likely harder to fit. Nevertheless, the fitted values for the gradient fields remain reasonable. Data for all three repeats are shown in Supplementary Material Figure S1.

## 5. Magnetic field compensation

In our second demonstration, the aim was to show that we could not only accurately measure the amplitude of a DC magnetic field and its field gradient using the model, but also that we could feedback these fitted data to the bi-planar coils and consequently null the measured field; this would enable suppression of artefacts caused by head movement.

### 5.1. Method

The participant sat inside the MSR wearing the additively manufactured rigid helmet containing 40 OPMs, and the internal walls of the room were degaussed. Following this, dynamic stabilisation was activated. The unknown remnant DC magnetic field inside the MSR was then mapped, again with the participant completing a sequence of rotations and translations of the head to sample the field variation. Once the field had been mapped, and coefficients calculated, the seven coil currents required to best produce the equal and opposing magnetic field were found using the coil calibration matrix. These currents were then applied to the bi-planar coils, as outlined in Section 3.3.

After this first nulling process was completed, the magnetic field that remained was then mapped again, the coefficients calculated and updated currents applied to the coils to test whether this field could be further reduced. Finally, field mapping was performed once more to determine the coefficients of the magnetic field achieved by this further compensation. Two participants took part in the experiment and each completed five independent experimental runs.

In order to assess whether or not the remnant magnetic field had been reduced, we employed two summary measures. The first was simply the magnitude of the fitted coefficients of magnetic field before and after the nulling procedure; we expected these to decrease. To provide simple representative values, we calculated both the Euclidean norm of the uniform field vector and the Euclidean norm of the field gradient coefficients. The second measure was the standard deviation of the measured magnetometer data. Here we reasoned that, assuming head movements were similar before and after nulling, and that the measured fields were dominated by the movement artefact, the standard deviation of the measured fields should be reduced following nulling. Standard deviation of the measured field variation was calculated for the eight OPMs involved in field mapping, and the result averaged across sensors.

We also calculated the Pearson correlation coefficient between the measured magnetometer data and the model fit; note that values close to 1 represent high confidence that the model was fitting the data accurately, and consequently that the magnetometer data were dominated by the motion artefact. Finally, we took data from the OptiTrack camera and calculated the standard deviation of the motion parameters (3 rotation and 3 translation) in order to summarise the extent to which the two participants moved, and the similarity of that movement across experimental runs.

### 5.2. Results

**Fig. 4.**
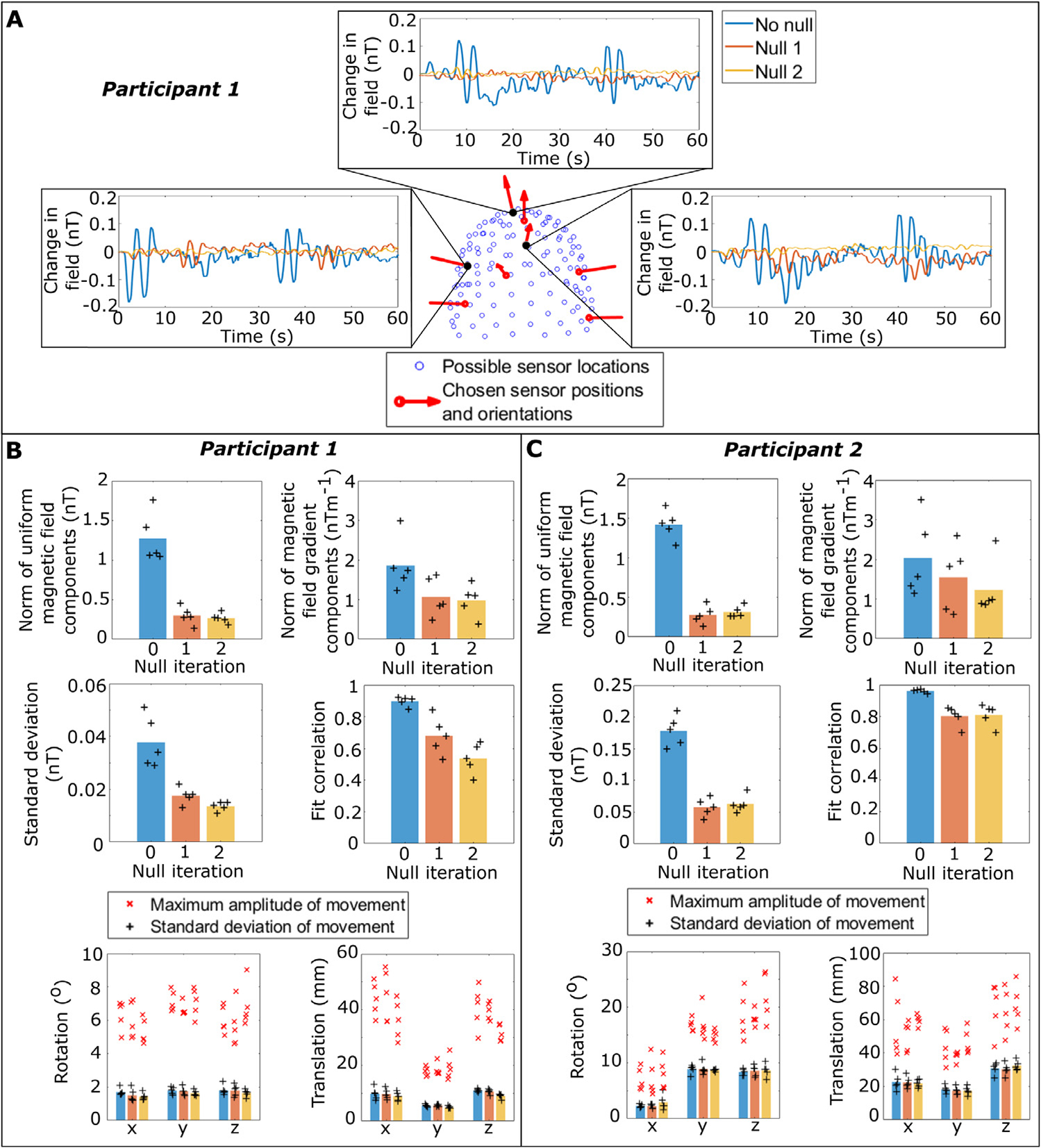
Field nulling A) Example magnetic field data measured from three selected OPMs during head motion. The OPM locations are shown on the central panel in black; red arrows show sensor orientations. In the field plots, measured fields prior to nulling are in blue. Fields after one null are shown in orange and after two nulls are shown in yellow. Deflections in these data are largely the result of movement in the background field. Notice how this movement artefact is diminished after nulling. B) Summary of results for participant 1: The upper panel shows the norm of the uniform magnetic field (left), and norm of the magnetic field gradients (right) before and after nulling. The bars represent the mean value across five repeat experiments, and the individual data points are shown as black crosses (+). The centre panel shows the standard deviation of the field measurements (left) and correlation to the model fit (right) again before and after nulling. Finally, the lower panels show movement parameters (left and right panels show rotation and translation, respectively). Here, the black crosses show the standard deviation of movement from the equilibrium (mean) position in each experiment, and the mean value is given by the bars. The red crosses (x) show the maximum movement recorded in each experiment. C) Equivalent to (B) for participant 2. (For interpretation of the references to colour in this figure legend, the reader is referred to the web version of this article.)

Fig. 4A shows an example data set; data acquired during movement are shown from 3 representative OPMs, with their locations in the helmet given in the central panel. The blue, orange and yellow lines respectively, show data prior to nulling, after the first nulling currents were applied, and after the second, updated currents were applied. As expected, the variance of the measured signal decreases, suggesting that the nulling procedure is reducing the effect of movement in a background magnetic field. In this single example, the fitted uniform magnetic field magnitudes were 1.42 nT before nulling, 0.46 nT after the first null and 0.27 nT after the second null. The norm of the magnetic field gradients was estimated as 1.79 nTm^−1^ before nulling, 1.52 nTm^−1^ after the first null and 0.84 nTm^−1^ after the second null.

Fig. 4B and C show the results across all five runs for participants 1 and 2, respectively. In both Fig. 4B and C, the upper left bar chart shows the Euclidean norm of the three uniform magnetic field components before and after nulling, averaged over experimental runs (individual data points for each experiment are given as black crosses). The average norm of the magnetic field gradient components (not including *α*_8_) is shown in the upper right bar chart. For participant 1, the Euclidean norm of the uniform field was reduced from 1.3(+0.5, −0.2) nT to 0.27(+0.09, −0.08) nT (mean and range of measured data), representing a 13 dB reduction. For participant 2, the uniform field was reduced from 1.4(+0.2, −0.3) nT to 0.31(+0.11, −0.05) nT; also a 13 dB reduction. Reduction of the gradients was less dramatic: we estimated that the norm of the gradient fell from 1.9(+1.1, −0.6) nTm^−1^ to 1.0(+0.5, −0.6) nTm^−1^ after two nulls in participant 1, and from 2.0(+1.5, −0.9) nTm^−1^ to 1.2(+1.2, −0.37) nTm^−1^ in participant 2.

In agreement with these data, we saw a similar drop in the standard deviation of the time-courses of the magnetometer data. The centre left panels of Fig. 4B and C show the standard deviation averaged across sensors (given by the crosses) and then across experimental runs (given by the bars), before and after nulling for each participant. The average standard deviation of the artefact was reduced from 38(+13, −9) pT to 14(+2, −3) pT after two nulls for participant 1 and 178(+32, −28) pT to 63(+22, −14) pT for participant 2 (note here that movement was more extensive for participant 2, hence the larger recorded artefact).

The centre right bar charts in Fig. 4B and C show the correlation to the magnetic field model; values close to 1 indicate high confidence in the fit. These values were averaged across sensors (shown by the crosses), and then across runs (shown by the bars). As might be expected, we observe a decrease in correlation as the field is nulled; this likely reflects the fact that the magnetometer data is becoming dominated by sources of artefact other than movement. Finally, the lower panels in Fig. 4B and C show the range of rotations and translations performed by the participant during each field mapping procedure. The bars show the standard deviation averaged across the five experiments; the red crosses show the maximum movement in each experiment. Importantly, whilst the two participants carried out different movements, those movements were similar across repeats for the individual participants.

## 6. MEG demonstration

The final experimental demonstration aimed to show that, in a real MEG experiment, our nulling process would reduce the effect of motion artefact and therefore improve the quality of MEG data recorded in the presence of participant movement. To this end, we undertook a visual steady-state evoked response experiment.

### 6.1. Method

Two participants took part in the experiment. The participant was seated inside the MSR, ~80 cm from a back-projection screen. The visual stimulus comprised a centrally-located green square on a black background, which was presented by projection, with a visual angle of 9° In a single trial, the square flashed at 6 Hz for 10 s, preceded by a 10 s rest period in which only a black screen was shown. This was repeated 25 times giving a total experimental length of 500 s. We expected to measure a driven, 6 Hz steady-state response in occipital sensors during the active periods.

Again, the participant wore the additively manufactured helmet containing 40 OPMs. Once the helmet was mounted, the MSR door was closed and the internal walls degaussed. During the recording, the participant was instructed to focus on the square whilst performing head movements, such as nods and shakes of the head. The experiment was first performed without background magnetic field compensation (the norm of the uniform magnetic field components was 1.25 nT for participant 1 and 1.40 nT for participant 2). Our nulling procedure was then applied, and the experiment repeated (participant 1: 0.13 nT, participant 2: 0.46 nT). Participant movements were monitored throughout using the OptiTrack camera, in order to assess the equivalency of movement during the first and second (pre- and post-nulling) runs.

We expected the magnetic field artefacts generated by continuous head movements to manifest at low frequencies, with the majority of the interference between 0 and 2 Hz. A 6 Hz flicker frequency was chosen such that the response would not be masked by movement artefact in the case where no field nulling was applied. This would enable comparison of the magnitude of the low frequency movement artefact with and without our field mapping and nulling method. We also hypothesised that the 6 Hz peak would have a larger SNR when data were recorded with the field nulling procedure applied.

Raw OPM data, collected at 1200 Hz, were filtered by a low-pass, finite impulse response filter of order 50 with a 10 Hz cut-off frequency. These time-series data were segmented into individual trials and then averaged in the time domain, prior to computation of the fast Fourier transform (FFT) of this averaged trial. The absolute value of the FFT was taken (1/10s = 0.1 Hz frequency resolution) and scaled to units of femtotesla to produce amplitude spectra. Separate spectra were derived for the stimulus ‘on’ and ‘off’ periods; this analysis was applied to each channel separately and an estimate of the SNR (defined as the height of the 6 Hz peak during the on period, divided by the height of the 6 Hz peak during the off period) was calculated for all channels.

### 6.2. Results

**Fig. 5.**
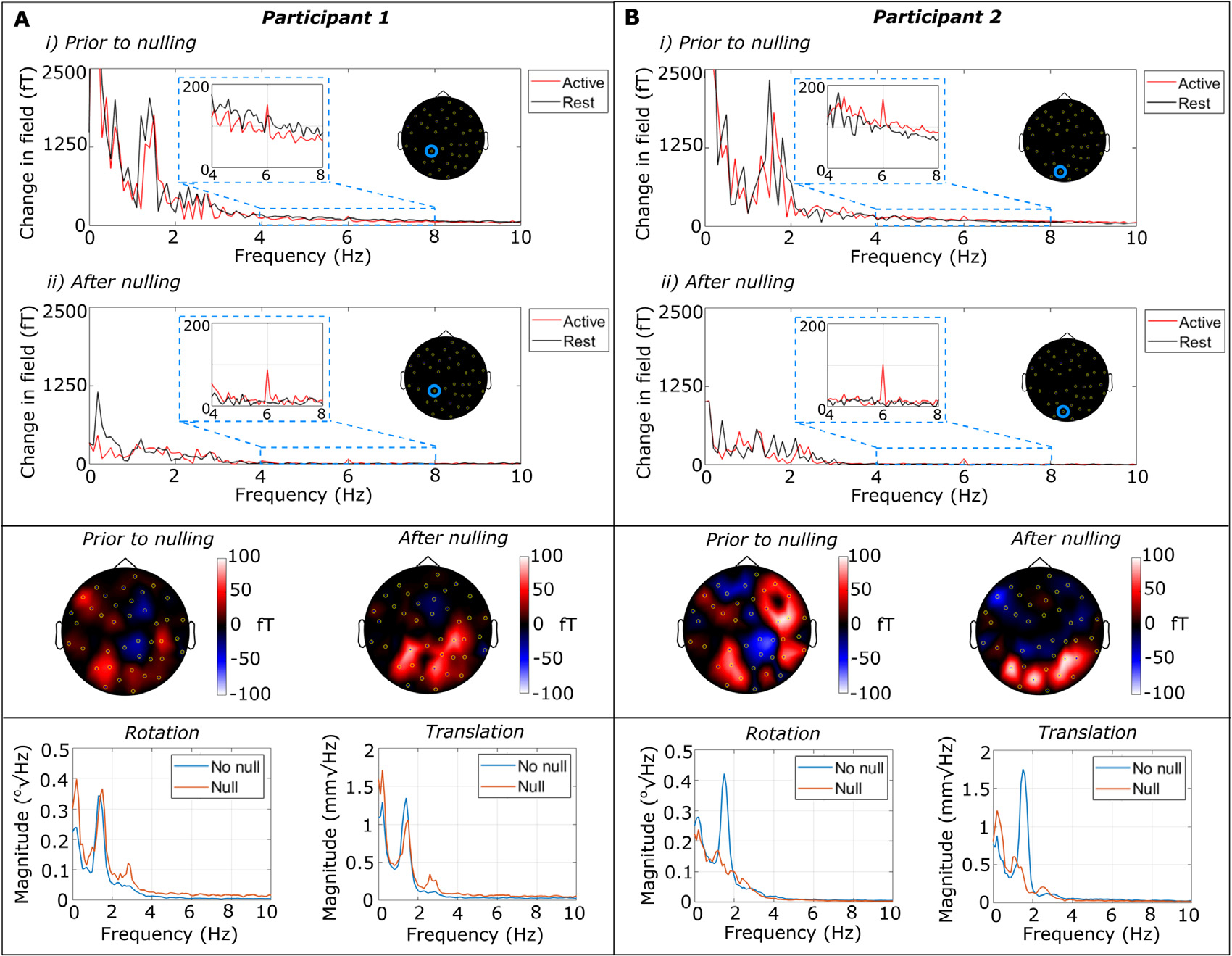
MEG demonstration. . A) MEG data from participant 1. B) Equivalent data from participant 2. In both cases: upper panel – amplitude spectral density derived from trial-averaged data taken from the single channel with the most consistent 6 Hz response. Data from the active segment of the trial (i.e. when the stimulus is flashing) are in red and data from the resting segment are shown in black. Plot i) shows the case with no nulling applied and ii) shows the case with nulling. Notice the overall drop in low-frequency artefacts. The inset graphs show the same data, but focussing on the 4–8 Hz range (i.e. where we expect to see a 6 Hz neural response to the stimulus). In all cases the response is clear, but is more prominent when field nulling is applied. Centre panel – spatial topography of the difference in 6 Hz signal strength between stimulus on and off, plotted for all sensors. Note that we expect the strongest response in sensors covering the visual areas. Lower panel – power spectra of movement data showing rotations (left) and translations (right). Data without nulling shown in blue, and with nulling in orange. (For interpretation of the references to colour in this figure legend, the reader is referred to the web version of this article.)

Fig. 5A shows results for the first participant, and Fig. 5B shows equivalent results for the second participant. In both cases, the upper panel shows the amplitude spectra computed for the active and rest periods separately, taken from the OPM that exhibited the largest, and most consistent, 6 Hz peak (the location of this sensor is marked on the inset diagram). Plot i) shows the case with no field nulling and ii) shows data with the bi-planar coils activated. Notice that significant artefacts are observed in both cases that manifest at very low (<2 Hz) frequency. However, these artefacts show a marked reduction in the case where field nulling was applied – providing evidence that they are generated by head movement in the background field. In the 0–2 Hz band, these data suggest that nulling affords a five-fold reduction of interference for participant 1, and a four-fold reduction for participant 2 (in both cases calculated as the integral of the signal without nulling between 0 and 2 Hz, divided by the equivalent integral with nulling). This is consistent with the decrease in field that we might expect from Fig. 4.

The two inset Figures in the upper panel show a zoomed-in plot of the 6 Hz peak. This peak is observable in all cases, but becomes more prominent after field nulling. Quantitatively, the SNR of the 6 Hz peak increased from 1.2 to 9.9 in participant 1, and from 1.6 to 11.0 in participant 2. We note, however, that the amplitude spectra corresponding to the experiments without field nulling feature a higher baseline, which increases the amplitude of the 6 Hz peak compared to the case with nulling applied. Specifically, linear trends present in the average trial time-course of the magnetometer data were also present in the average trial time-course of the motion data for each participant, suggesting these trends result from movement of the sensor through a non-zero magnetic field. The Fourier transform of these linear trends produce a 1/frequency contribution that interferes constructively with the 6 Hz neuronal peak and raises the baseline of the measurement (see Supplementary Material). Our field nulling technique does not affect the physiological signal strength at 6 Hz, but reduces the motion artefact that causes this constructive interference.

The data in the upper panel are from a single sensor. In order to get a more global picture of the improvement in data quality, the central panel shows sensor-space topographies detailing the difference in the magnitude of the 6 Hz peak between the active and rest periods, for all sensors. Given the nature of the stimulus, we would expect this difference to be most prominent in sensors covering the occipital lobe. This is largely the case, however a more focal response is observed in the case where field nulling is employed, suggesting that the spatial topography of the 6 Hz response is being degraded by the presence of the movement artefacts in the data.

Finally, the lower panels show the power spectral density (PSD) of the rotations (left) and translations (right) of the helmet recorded by the OptiTrack camera. The case with no field nulling is shown in blue and the case with field nulling is shown in orange. In participant 1, the data show similarly sized movements, with largely equivalent power spectra with and without nulling. For participant 2, whilst the magnitude of movement was largely similar, the frequency components post-nulling were missing the large peak at ~1.7 Hz. This is a potential confound since the participant clearly did not carry out the same movements in the two experiments.

## 7. Discussion

Recent years have shown that OPMs have the potential to revolutionise MEG as an imaging technology, with the promise of a wearable system that adapts to any head shape (Hill et al., 2019), enables movement during scanning (Boto et al., 2018), and provides higher sensitivity (Boto et al., 2017) with improved spatial resolution (Boto et al., 2019). However, this potential can only be realised if background magnetic fields can be appropriately controlled, and this proves a significant challenge. In this work, we have shown that even in an OPM-optimised MSR with extremely low (<2 nT) remnant fields, head movement generates significant artefacts, which manifest as low-frequency interference that obfuscates the MEG signal. To counter this effect we have introduced a field mapping technique in which participants move their head to sample background magnetic fields using scalp-mounted OPM sensors; resulting data are compared to simulations in order to derive coefficients representing the three uniform magnetic field components and five magnetic field gradient components in the vicinity of the head inside the MSR. We were able to show that this technique accurately reconstructed the magnitude of known offset fields. Moreover, by feeding these coefficients into a bi-planar coil system (Holmes et al., 2019, 2018) via a coil calibration matrix, we were able to reduce the uniform magnetic field from a magnitude of 1.3 ± 0.3 nT, to 0.29 ± 0.07 nT (mean ± standard deviation across both participants). This extremely low field is similar to other high performance magnetically-shielded environments (Altarev et al., 2014; Bork et al., 2001). Most importantly, we have shown that this magnetic field compensation, which was readily implemented in combination with dynamic stabilisation of temporal variations of the uniform field components, significantly reduces the motion artefact present in OPM-MEG data. Low-frequency artefact (0—2 Hz) was found to be reduced by a factor of five in a visual steady-state evoked response experiment using 6 Hz stimulation. We therefore suggest that this technique could be used in future OPM-MEG experiments to significantly improve the quality of data, especially in paradigms that encourage head movement.

Our previous OPM-MEG studies involving substantial participant movement (Boto et al., 2018; Hill et al., 2019; Roberts et al., 2019) were performed within a MSR that has a remnant magnetic field of ~30 nT (Vacuumschmelze, Hanau, Germany), and houses a cryogenic MEG system. The efficacy of coil systems to reduce MSR background magnetic fields in the range of 20–70 nT to <1 nT has been reported in previous papers (Borna et al., 2019; Holmes et al., 2019; Iivanainen et al., 2019). However, a MSR was recently installed at our institution with a comparatively lower remnant magnetic field of <2 nT, which is achieved by regular use of integrated degaussing coils (MuRoom, Magnetic Shields Ltd., Kent, U.K.). Equivalent demagnetisation of the walls of a more conventional MEG MSR may achieve a similarly low remnant magnetic field (Voigt et al., 2013), however the required coils are not typically included during MSR installation, and fitting such coils to a room that has already been built would pose a significant challenge. Therefore, the commercial availability of a MSR that can readily provide such a low background magnetic field has positive implications for the future of OPM-MEG, however using electromagnetic coil systems to further improve upon this already low background magnetic field becomes more challenging.

In order to further suppress such a low remnant magnetic field, a high accuracy of characterisation of the field and its spatial variation is required. Previously, field mapping for OPM-MEG has been achieved using stationary reference arrays positioned close to (e.g. Borna et al., 2020) or on the head (e.g. Iivanainen et al., 2019). Our previous reference array approach sampled the full vector field at two locations behind the participant’s head, yielding measurements of the three uniform magnetic field components and three of the five magnetic field gradients (Holmes et al., 2018). Increasing the number of reference sensors and sampling at more locations would improve this method, however it would also further enclose the scanning environment and so reduce the volume available for participant movement. Our new technique for mapping the remnant magnetic field via head movement has two distinct advantages: firstly, head-mounted OPMs sample the background magnetic field, meaning that the mapping is performed in the precise region encompassing the participant’s head (rather than behind the head at the location of the reference array, for example). This makes the nulling more accurate. Secondly, in order to sample the remnant field, the OPMs are not required to measure an absolute field, but rather movement-induced field change. This leads to a more accurate characterisation, since measurement of absolute field by an OPM (or indeed any magnetometer) is often distorted, for example by small amounts of magnetisation in the sensor itself, which can lead to field offsets. The improvement of our present approach over our reference array findings can be quantified; in our previous approach, we achieved a magnetic field of 0.74 nT: rotation of a magnetometer by 10° in this field will generate an artefact of amplitude 0.14 nT. Rotation by the same amount in the magnetic field of 0.29 nT achieved here will generate a change of 0.055 nT – a reduction in artefact size by a factor of 2.5.

Despite offering improved characterisation of the remnant magnetic field for OPM-MEG, our head-movement based sampling technique does pose some disadvantages that should be considered. Firstly, we have assumed that the measurements made by the OPMs during the head movement procedure are dominated by motion artefacts. Theoretically, this should be the case (e.g. brain activity is much smaller (and typically higher frequency) than the movement artefact and so should not impact the fitted data). However, at the time of writing there is a known problem with interference due to the cables used in the QuSpin sensors – specifically that relative movement of the OPM cables generates low-frequency artefacts that correlate with the movement data. Whilst this problem will be corrected in future revisions of the cables, at present it is a source of error that likely contributes to inaccuracies in the fit coefficients (and consequently the efficacy of the nulling procedure). Second, the field nulling process is extremely sensitive to the accuracy of the measured sensor positions and orientations. Here, we employed an OptiTrack Duo camera placed in front of the participant, and tracked the movement of six markers on the front of the helmet relative to six markers that were placed on the coils. Whilst this approach works well, it is possible that improved motion tracking would yield more accurate data. Specifically, since the OptiTrack Duo has only two cameras, the field of view was limited to markers on the front of the helmet. Any inaccuracy in characterisation of these markers at the front of the head would likely be amplified at the back of the head, resulting in degradation of the quality of the fit to data acquired towards the rear of the head. Additionally, the limited field of view meant that reference points could only be placed on a single coil. Any slight inaccuracy in coil placement could then generate a systematic error in reconstructed head position. These inaccuracies in head tracking may have led to errors in the obtained coefficients of magnetic field. An improved optical tracking system with multiple cameras placed around the participant would expand the field of view, enabling tracking of markers more evenly spread across the helmet as well as additional stationary reference points. This would likely lead to an improved accuracy of field characterisation and consequently improved field nulling.

The significant problem of field drift was ameliorated by the use of dynamic stabilisation. Specifically, a reference array was used to measure temporal changes in the three uniform magnetic field components. These dynamic changes were then fed back to the coils in order to generate equal and opposite field shifts. This allowed us to ‘lock’ the fields such that field drift over the course of an experiment, and the remnant DC field inside the MSR, could be separated. In other words, it allowed us to remove temporal field variation prior to mapping, and nulling the DC field. This was an essential step in order to achieve the low fields shown in Fig. 4, and the reduction of motion artefact in MEG data shown in Fig. 5. Our dynamic stabilisation procedure is effective: it allows magnetic fields to remain stable to within 0.2 nT over a time period of 20 min (see Appendix B). Again, however, there is some room for improvement in future iterations of the technique. Specifically, in the present work we only dynamically stabilised the three uniform magnetic field components; if we were to also dynamically stabilise magnetic field gradients, this would likely lead to more accurate characterisation of the parameters in the model relating to gradient fields, and consequently lead to more accurate nulling. Possibly the use of a more extensive reference array might be of some benefit, or even incorporating the dynamic stabilisation into the sensors on the helmet (Iivanainen et al., 2019) (though this would require separation of the field change due to movement, and that due to drift).

Despite some of the problems mentioned above, the final remnant magnetic fields that we were able to achieve using this method were extremely low. This resulted in MEG data in which motion artefact was reduced five-fold by application of our technique. This, we believe, will be of significant importance if we are to move forward and realise the potential of a wearable MEG system. Head movement typically manifests at low frequency, and so the technique reported is likely to become important in studies of either sustained responses (DC field shifts for the duration of stimulation), delta, or theta oscillations. Given the apparently important role of frontal midline theta oscillations in cognition (e.g. (Brookes et al., 2011)), and the purported clinical relevance of delta oscillations in, for example, patients with mild traumatic brain injury (Huang et al., 2017), it is likely that removal of head movement artefacts will become increasingly important for OPM-MEG. In addition, whilst in healthy participants head movement artefacts may be low-frequency, in patients (e.g. an epilepsy patient suffering a seizure) or in specific participant groups (e.g. infants or patients with movement disorders), it is conceivable that head movement may begin to manifest at higher frequency, where it could overlap with alpha or beta oscillations, for example. Here again, the importance of suppressing this effect at source by optimally minimising the background magnetic field will be of great importance.

Finally, there are other refinements that could be made to improve this technique. Firstly, we have concentrated on spatially-uniform magnetic field and linear magnetic field gradient terms, but there is no reason why the model cannot be extended to fit higher-order spherical harmonics. Here, we limited the fit because our coil array was confined to only generating uniform magnetic fields and magnetic field gradients. However, new types of coil design and the inclusion of higher-order terms have the potential to offer significant improvements. Secondly, the method of coil calibration that we used was somewhat limited to a data-driven approach. However, if the interactions between the magnetic fields generated by our coils and the mu-metal walls of the MSR are properly taken into account (e.g. via appropriate evaluation of the boundary conditions imposed on the magnetic field by high-permeability materials (Holmes et al., 2019; Packer et al., 2020; Zetter et al., 2020)) it is possible that improved coil calibration may make nulling more effective. Finally, from a practical point of view, the nulling procedure could be made quicker via the use of less data, and by more detailed instructions of how to carry out the head motion. This may become important, particularly for some participant groups who may find it difficult to carry out the series of head translations and rotations required.

## 8. Conclusion

In this paper we present a new way to map background magnetic field and magnetic field gradient, using data sampled by an on-scalp sensor array as a participant moves their head through the remnant magnetic field. By feeding back the fit coefficients of magnetic field to an electromagnetic coil array, we were able to effectively minimise this magnetic field. Results show that we can null the field inside an OPM-optimised MSR to a level of <0.3 nT. This, in turn, offers a marked reduction in the motion artefact present in OPM-MEG data. This method will be important in future studies where OPM-MEG is used, particularly when measuring neuromagnetic effects at low frequency, and also in cases where natural head movement is encouraged.

## Supplementary Material

Supp

## Figures and Tables

**Fig. A1.**
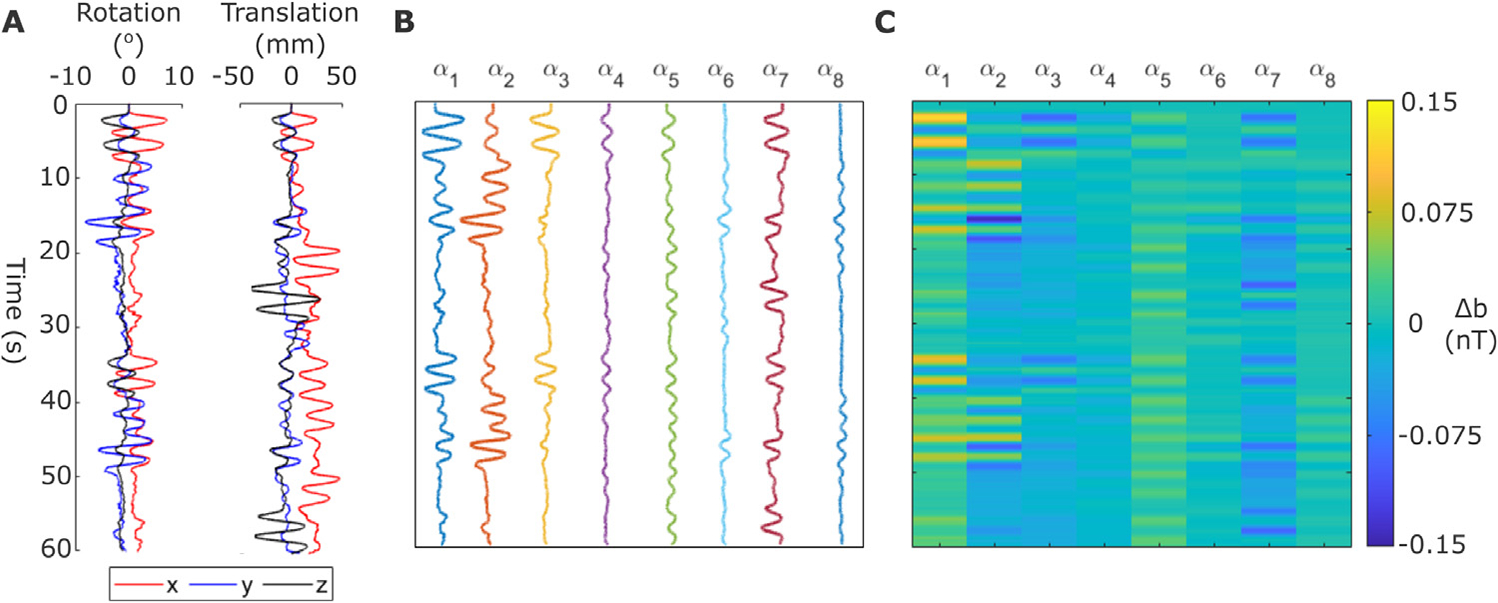
Visualisation of B_mat_ for a single sensor. *A) The measured rotations and translations of a single sensor, relative to its initial orientation and position. B) In the case of a single sensor, each column of* **B**_mat_ *corresponds to the change in measured field due to movement through a unit contribution in each of the 8 magnetic field or magnetic field gradient components in our model. C) Image representation of the corresponding matrix,* **B**_mat_.

## Data Availability

Data are available from the corresponding author on request, under a data sharing agreement. Some aspects of code are commercially sensitive, they can be shared with academic partners under a non-disclosure agreement.

## Appendix A. *B*_mat_ matrix

The matrix, **B**_mat_, was constructed to characterise the change in magnetic field predicted by our model due to movement through a 1 nT or 1 nTm^−1^ magnetic field or magnetic field gradient in each component, respectively. By evaluation of the pseudo-inverse of this matrix, the coefficients that best characterised the remnant magnetic field inside our MSR could be determined in accordance with the following equation:

(A1)
$$B_{\text{mat}}\alpha =b,$$

where ***α*** is a column vector containing the eight coefficients of magnetic field and ***b*** is the target field column vector, which contains the magnetic fields measured by the OPM array. Here we describe the construction of **B**_mat_ in more detail.

Using the rotation and translation data for each sensor obtained during the field mapping process Fig. A1A), we simulate the change in magnetic field measured by each sensor as it moves through a background magnetic field consisting of a unit contribution in one component of our model. The measured field is calculated by evaluating Eqs. (7) and ((8) for each sensor at each time-point, using the measured sensor position, (*x, y, z*), and orientation, ***e***. This is repeated for each of the eight components in our model.

We construct the matrix, **B**_mat_, such that it has eight columns, each corresponding to a component of magnetic field or magnetic field gradient in our model. Each row corresponds to one sensor at one time-point, and contains the modelled change in magnetic field for each component. In the case of a single sensor, each column of **B**_mat_ forms a modelled sensor time-course, which characterises the change in measured field experienced by that sensor due to its movement through the corresponding unit magnetic field or field gradient component, as shown in Fig. A1B. The corresponding **B**_mat_ for this sensor is shown in Fig. A1C.

For an array of *N* OPMs whose movements are measured over *T* time-points, **B**_mat_ has *T* · *N* rows. The target field column vector, ***b***, also has *T* · *N* rows while the column vector of coefficients, ***α***, is unchanged. These are arranged as follows:

(A2)
$$B_{\text{mat}}=\left(\begin{array}{ccc} B_{1,1,\alpha_{1}} & \cdots & B_{1,1,\alpha_{8}}\\ \vdots & \ddots & \vdots \\ B_{1,N,\alpha_{1}} & \cdots & B_{1,N,\alpha_{8}}\\ \vdots & \ddots & \vdots \\ B_{T,N,\alpha_{1}} & \cdots & B_{T,N,\alpha_{8}} \end{array}\right),\;\alpha =\left(\begin{array}{c} \alpha_{1}\\ \vdots \\ \alpha_{8} \end{array}\right),\;b=\left(\begin{array}{c} b_{1,1}\\ \vdots \\ b_{1,N}\\ \vdots \\ b_{T,N} \end{array}\right).$$

The coefficients of magnetic field were found using the pseudo-inverse of **B**_mat_. If the participant made no movements at all then the matrix would be singular, but in practice, with proper instruction, this is very unlikely.

## Appendix B. Dynamic stabilisation

In order to ensure accurate mapping of the remnant magnetic field inside the MSR it was necessary to dynamically stabilise the uniform components of the field during experiments. Here we provide a simple demonstration to highlight the importance of dynamic stabilisation.

An array of 40, second-generation QuSpin OPM sensors were placed inside the rigid helmet, which was positioned in the MSR at the centre of the bi-planar coils and close to a reference array containing three OPMs. The position and orientation of the reference sensors with respect to the head and the coils is shown in Fig. B1A. The internal mu-metal walls of the MSR were degaussed and ‘empty room’ magnetic field data were recorded for 20 min. The experiment was repeated with and without dynamic stabilisation.

Fig. B1B shows sensor time-course data collected from the OPM array when dynamic stabilisation was not used (left), and the equivalent data when dynamic stabilisation was active (right). Figure B1C shows the median value (across sensors) of the power spectral density (PSD, computed by segmenting time-course data into 10 s chunks and applying a flat-top window before taking the fast Fourier transform of each chunk and averaging the results). Results are shown with dynamic stabilisation switched off (red) and active (blue). Fig. B1D shows an estimate of the field attenuation of the dynamic stabilisation system as a function of frequency (obtained via division of the two PSDs). The field attenuation is expressed in decibels, where a positive value indicates shielding.

Visual inspection of the data in Fig. B1B shows that the temporal field drift at low frequencies is of the same order as the static background field following degaussing inside the OPM-optimised MSR. More quantitatively, the maximum change in magnetic field over the 20-minute recording was 980 pT; given the expected static background of ~2 nT, this could lead to errors in the magnetic field model of around 50%. A qualitative comparison of the plots in Fig. B1B suggests that temporal variations in the field occurring over timescales of ~10 s are well compensated by the dynamic stabilisation. Indeed, when dynamic stabilisation was switched on, the maximum field change over the 20 min recording was reduced to 214 pT, and consequently the error on nulling would fall to around 10%. This confirms the critical need for dynamic stabilisation.

However, it is also clear from these results that dynamic stabilisation is not perfect. A steady drift in field remains even with dynamic stabilisation, albeit with a shallower temporal gradient. This effect may be due to changing field gradients within the room that are poorly sampled by the reference array and are therefore not compensated by the procedure. Consequently, it seems likely that improved reference arrays and dynamic nulling of field gradients might prove fruitful in improving nulling further. In addition, Figs. B1C and B1D show that, whilst for frequencies <1 Hz the dynamic stabilisation decreases interference, at higher frequencies interference is marginally increased. This is likely due to the bit-depth of the DACs used in the controller, and improved electronics, (e.g. separate controllers for the static and dynamic stabilisation schemes) could be used to reduce this effect.
