# Supplementary material for: Precision magnetic field modelling and control for wearable magnetoencephalography
Source: Neuroimage. Author manuscript; Available in PMC 2022 Jul 1. (PMC9248349; doi:10.1016/j.neuroimage.2021.118401)
Supplement: Supp [file NIHMS1805132-supplement-Supp.pdf]

Supplementary Material – Mapping Known Fields

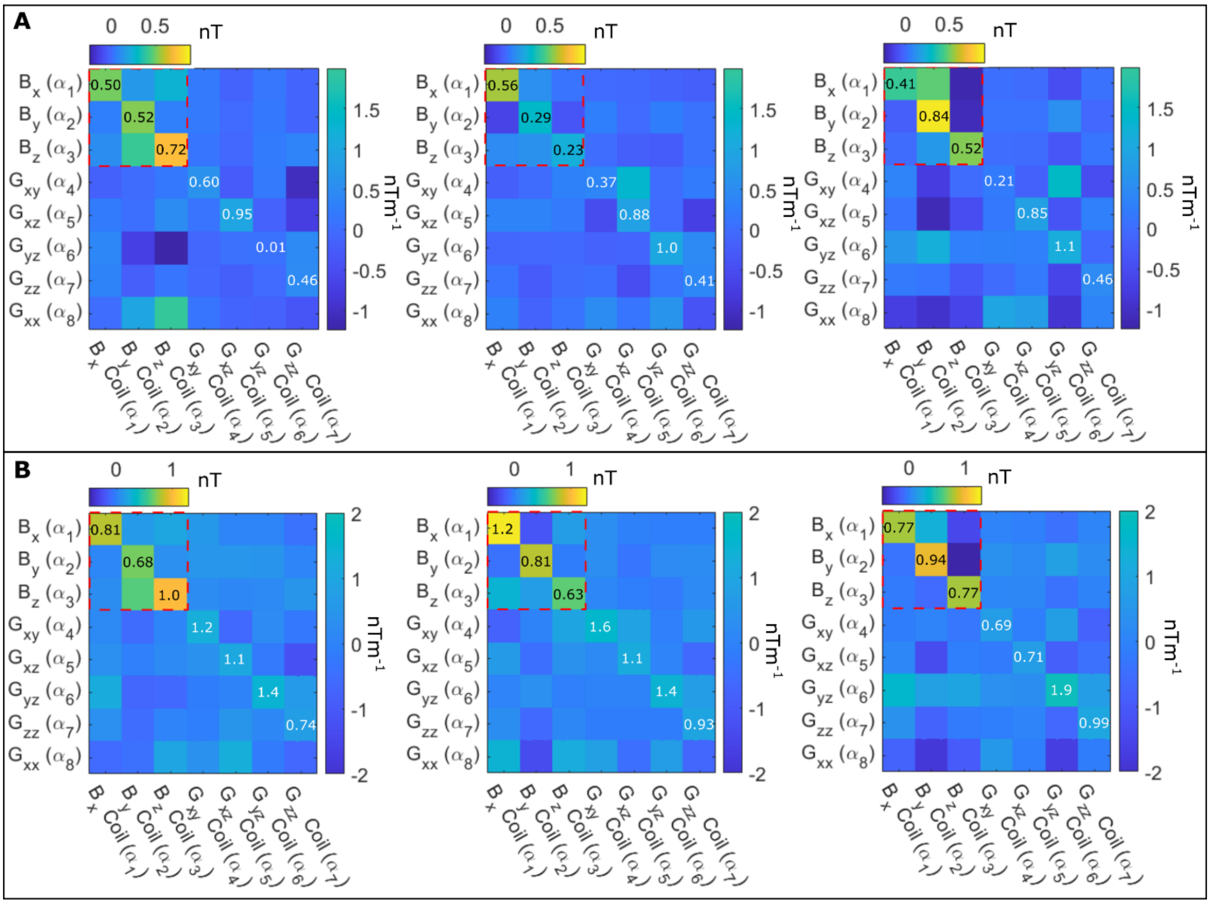

Figure S1 – All data for mapping known fields. *A) The results of mapping known fields for three repeat experiments: a known magnetic field of 0.5 nT, or a field gradient of 0.5 nTm^{-1} was generated by each coil. The values in the matrix show the model fit to those fields, i.e. the numbers along the diagonal should be close to 0.5. B) Equivalent for 1 nT or nTm^{-1} magnetic field or magnetic field gradient, numbers along the diagonal should be close to 1.*

Supplementary Material – MEG Demonstration

In the Results of our MEG Demonstration, we noted that the amplitude spectra corresponding to the experiments without field nulling featured a higher baseline, which increased the amplitude of the 6 Hz peak compared to the case with nulling applied. This is due to constructive interference between the 6 Hz response and the baseline artefact, resulting from linear trends in the time series data following trial averaging. These average time-courses show a negative linear trend during the resting period and a positive linear trend in the active period. These trends are also evident in the averaged time-courses of the recorded participant movements (see Figure S2).

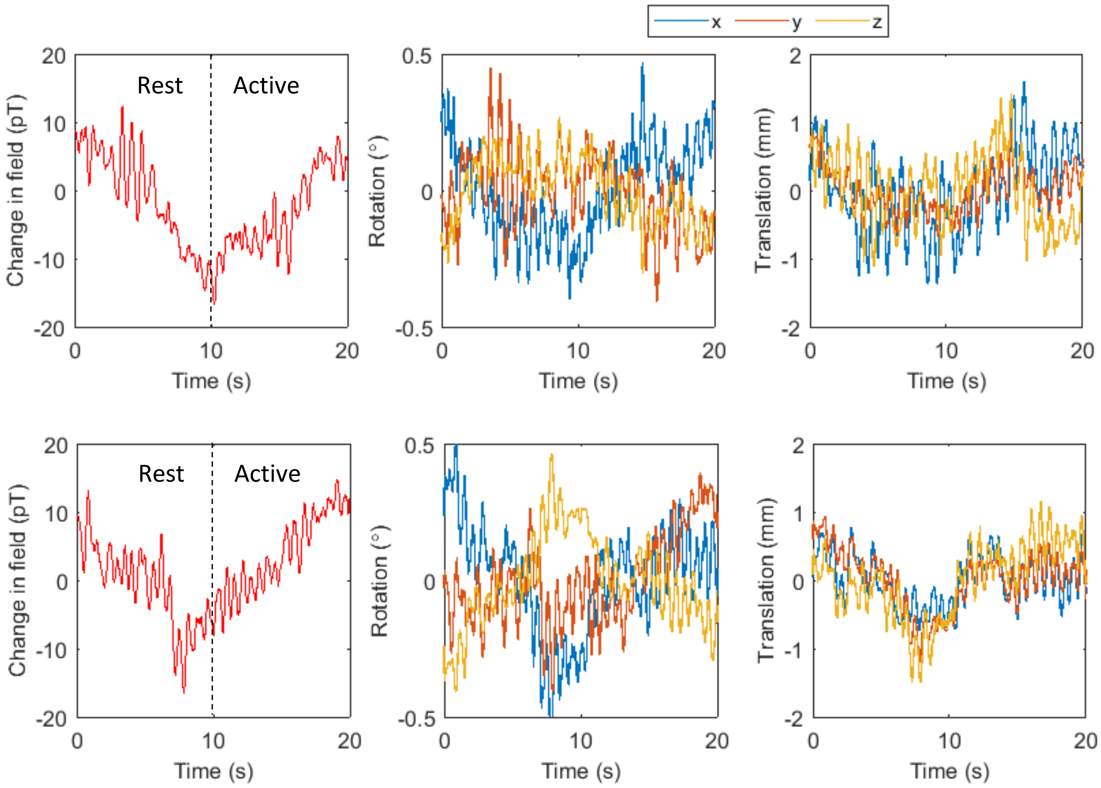

Figure S2: Average time-courses: *Left: Trial averaged magnetic field data from a single sensor (the same sensor used in the upper panels of Figure 5) acquired without field nulling. Note the approximately linear trends during the rest and active windows. Centre and right panels show participant rotation and translation respectively, also averaged across trials. Note again the clear linear trends, demonstrating that the origin of the trial averaged field change is likely to be participant motion. Upper panel shows participant 1; lower panel shows participant 2.*

To recap – for our analyses in Figure 5 – an average over 25 trials was calculated for each sensor in the time domain. We then computed the fast Fourier transform (FFT) of the averaged data. The absolute value of the FFT was taken and scaled to units of femtotesla, to produce the amplitude spectra. To understand the effect of the approximately linear drifts apparent in the data, it proves useful to consider an analytical Fourier transform.

Let us assume we have a linear function, S(t), as below.

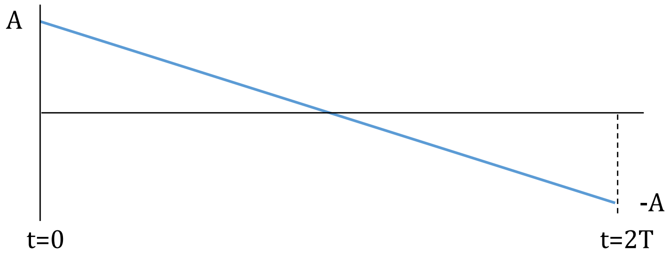

This function will be seen by the Fourier transform as

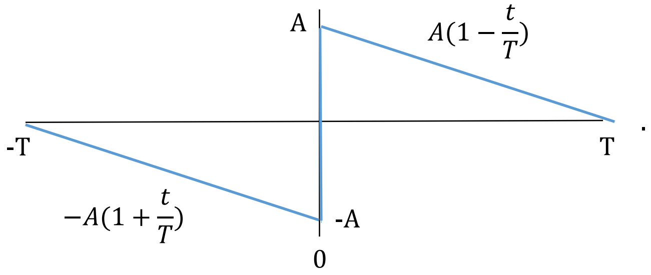

The Fourier transform of S(t) is given by

$$\mathrm{FT(S(t))}=\mathrm{A}\left[-\int_{-\mathrm{T}}^{0}\left(1+\frac{\mathrm{t}}{\mathrm{T}}\right)\mathrm{e}^{-\mathrm{i}\omega\mathrm{t}}\mathrm{dt}+\int_{0}^{\mathrm{T}}\left(1-\frac{\mathrm{t}}{\mathrm{T}}\right)\mathrm{e}^{-\mathrm{i}\omega\mathrm{t}}\mathrm{dt}\right], \quad \{S1\}$$

which can be written as

$$\mathrm{FT(S(t))}=\mathrm{A}\left[\int_{0}^{\mathrm{T}}\mathrm{e}^{-\mathrm{i}\omega\mathrm{t}}\mathrm{dt}-\int_{-\mathrm{T}}^{0}\mathrm{e}^{-\mathrm{i}\omega\mathrm{t}}\mathrm{dt}-\int_{-\mathrm{T}}^{\mathrm{T}}\frac{\mathrm{t}}{\mathrm{T}}\mathrm{e}^{-\mathrm{i}\omega\mathrm{t}}\mathrm{dt}\right]. \quad \{S2\}$$

Performing the integral we find

$$\frac{\mathrm{FT(S(t))}}{\mathrm{A}}=\left.\frac{\mathrm{e}^{-\mathrm{i}\omega\mathrm{t}}}{-\mathrm{i}\omega}\right|_{0}^{\mathrm{T}}-\left.\mathrm{e}^{-\mathrm{i}\omega\mathrm{t}}\right|_{-\mathrm{T}}^{0}+\left.\frac{\mathrm{t}}{\mathrm{i}\omega\mathrm{T}}\mathrm{e}^{-\mathrm{i}\omega\mathrm{t}}\right|_{-\mathrm{T}}^{\mathrm{T}}-\frac{1}{\mathrm{i}\omega\mathrm{T}}\int_{-\mathrm{T}}^{\mathrm{T}}\mathrm{e}^{-\mathrm{i}\omega\mathrm{t}}\mathrm{dt}, \quad \{S3\}$$

and evaluating the limits,

$$\frac{\mathrm{FT(S(t))}}{\mathrm{A}}=\frac{\mathrm{i}}{\omega}\left(\mathrm{e}^{-\mathrm{i}\omega\mathrm{T}}-1\right)-\frac{\mathrm{i}}{\omega}\left(1-\mathrm{e}^{\mathrm{i}\omega\mathrm{T}}\right)-\frac{\mathrm{i}}{\omega}\left(\mathrm{e}^{-\mathrm{i}\omega\mathrm{T}}+\mathrm{e}^{\mathrm{i}\omega\mathrm{T}}\right)-\left(\frac{\mathrm{e}^{-\mathrm{i}\omega\mathrm{T}}-\mathrm{e}^{\mathrm{i}\omega\mathrm{T}}}{\omega^{2}\mathrm{T}}\right), \quad \{S4\}$$

which simplifies to

$$\mathrm{FT(S(t))}=\frac{2\mathrm{Ai}}{\omega}\left[\frac{\sin\omega\mathrm{T}}{\omega\mathrm{T}}-1\right]. \quad \{S5\}$$

Taking the positive half of the spectrum, in the discrete Fourier transform we measure values at frequencies of $\mathrm{n}\Delta\omega$ for $\mathrm{n}=0{:}\frac{\mathrm{N}-1}{2}$, with $\Delta\omega=\frac{2\pi}{2\mathrm{T}}$. This means that

$$\mathrm{FT(S(t))}=\tilde{\mathrm{S}}(\mathrm{n}\Delta\omega)=\frac{2\mathrm{Ai}}{\omega}\left[\frac{\sin\mathrm{n}\pi}{\mathrm{nT}}-1\right] \quad \{S6\}$$

and so we find

$$\mathrm{FT(S(t))}=-\frac{2\mathrm{Ai}}{\omega}\left[\delta_{\mathrm{n},0}-1\right]. \quad \{S7\}$$

Therefore the Fourier transform of a linear change varies as $-\frac{2\mathrm{Ai}}{\omega}$, but goes to zero at $\omega=0$.

It is noteworthy that the linear trend, when Fourier transformed, results in a 1/frequency behaviour in the imaginary part of the signal. Conversely, the neuronal signal, which follows approximately a cosine function (due to the way the stimulus was delivered), results in a peak at 6 Hz in only the real part of the Fourier spectrum. This separation of the two effects into the real and imaginary components means that, when we compute the absolute value, the real and imaginary components sum in quadrature, such that the 6 Hz peak rides atop the 1/f baseline.

We reasoned that the constructive interference could be eliminated by de-trending the average trial time-courses. To do this, first order polynomial fits to the average time-courses in the rest and active periods were independently generated, and subtracted from the data (see Figure S3). This was done for data with and without field nulling applied.

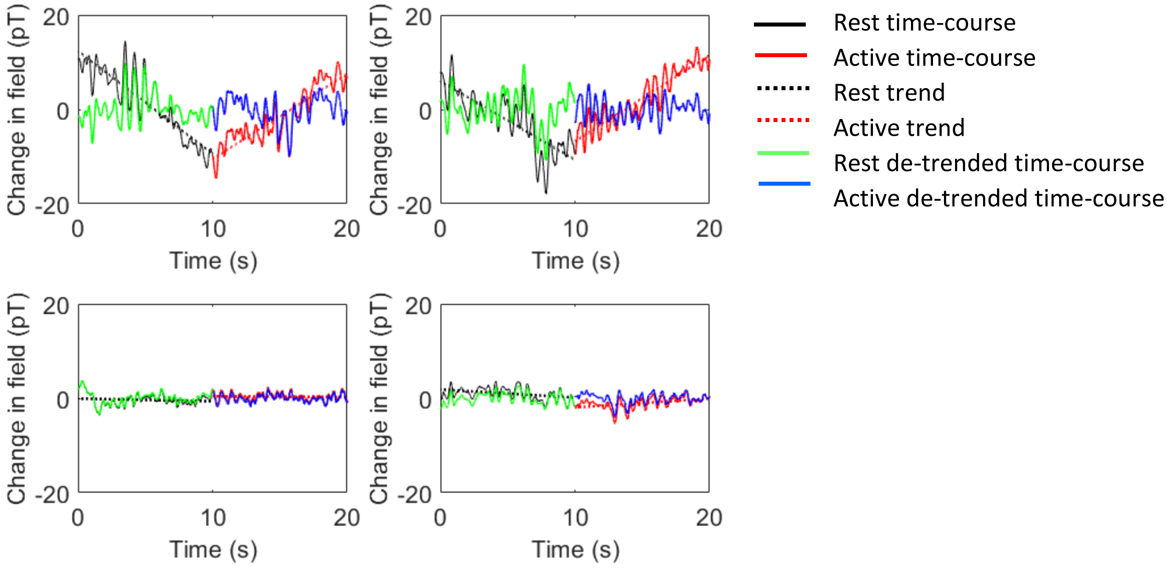

Figure S3: Fitting linear trends. *Two first order polynomials were fit to the data during the rest (0 < t < 10s) and active (stimulation) (10s < t < 20s) windows. These were then subtracted in order to de-trend the data. The four plots show this procedure in participant 1 (left) and participant 2 (right), both before (top) and after (bottom) nulling. In all plots, the original and de-trended data are shown overlaid, along with the linear fits.*

Following de-trending, the amplitude spectra were re-computed, and compared to the original amplitude spectra that were shown in Figure 5 of our manuscript. Figure S4 shows example spectra computed for the active window, with the original trial average shown in blue and the de-trended trial average in red.

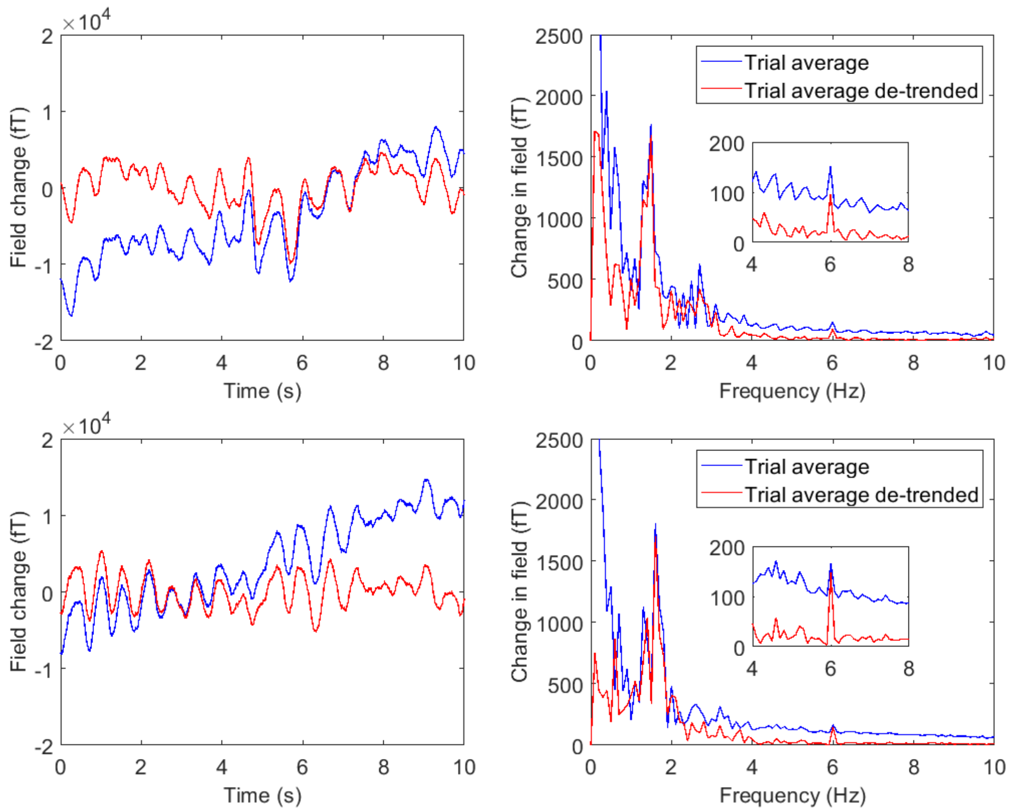

Figure S4: Fourier analysis of de-trended data. *Left hand plots show the average time-courses in the active window. Right plots show the corresponding Fourier amplitude spectra. The original data are shown in blue, and data with linear de-trending in red. Data obtained without field nulling is shown for participant 1 (top), and participant 2 (bottom).*

These results show that de-trending the average time-courses removes the 1/frequency contribution to the FFTs, preventing constructive interference with the signal of interest. We also recalculated the relative peak height due to stimulation (one measure of SNR – calculated as the peak height in the active period divided by the peak height at rest). In the de-trended data, for participant 1, the relative peak heights were 9.0 and 10.5 before and after nulling, respectively. For participant 2, these values were 11.2 and 9.6. These values show that the physiological signal strength at 6 Hz is largely unchanged by the nulling process, since following de-trending, the height of the 6 Hz peak is similar before and after nulling was applied.

Following de-trending, other low-frequency motion artefacts remain apparent, and are reduced by field nulling. Quantitatively, the integral of the FFT between 0 and 2 Hz is reduced by a factor of 3 by nulling, for both participants, in de-trended data. (This is distinct from a factor of 5 when no de-trending is used, showing that while de-trending is effective at removing the 1/frequency component it is not as effective at removing all motion artefact as field nulling.)

Discussion:

De-trending the trial average before computation of the FFT allows us to resolve the signal of interest with good SNR in the experiments, without field nulling applied. However, this approach is specific to this particular dataset in which the trends are linear and therefore easily removed. Such de-trending is unlikely to generalise to other OPM-MEG experiments, hence nulling the background magnetic field to minimise motion artefact and remove the problem *at source* is a more appropriate solution than post-processing. Furthermore, field nulling remains critical to OPM-MEG operation, since OPMs become insensitive to magnetic field if the change in field exceeds 5 nT. Sensor gain is reduced by as much as 5% following a 1.5 nT change in field, and 10% following a 5 nT change in field. Efforts (such as the field nulling method we present) to remove these artefacts at source are certain to benefit data quality.
